# Nitric Oxide Activatable Photodynamic Therapy Agents Based on BODIPY–Copper Complexes

**DOI:** 10.1021/acsptsci.4c00428

**Published:** 2024-12-10

**Authors:** Huriye Ilhan, Merve Şeker, Gülcihan Gülseren, Melike Ebrar Bakırcı, Ayşe İlayda Boyacı, Yusuf Cakmak

**Affiliations:** † Department of Biotechnology, Graduate School of Natural & Applied Sciences, Konya Food and Agriculture University, 42080 Konya, Turkey; ‡ Department of Molecular Biology and Genetics, Konya Food and Agriculture University, 42080 Konya, Turkey; § Department of Molecular Biology and Genetics, Necmettin Erbakan University, 42090 Konya, Turkey; ∥ Department of Metallurgical and Materials Engineering & Science and Technology Research and Application Center (BITAM), Necmettin Erbakan University, 42090 Konya, Turkey

**Keywords:** photosensitizer, boron dipyrromethene, fluorescence imaging, photodynamic cancer therapy, reactive oxygen species

## Abstract

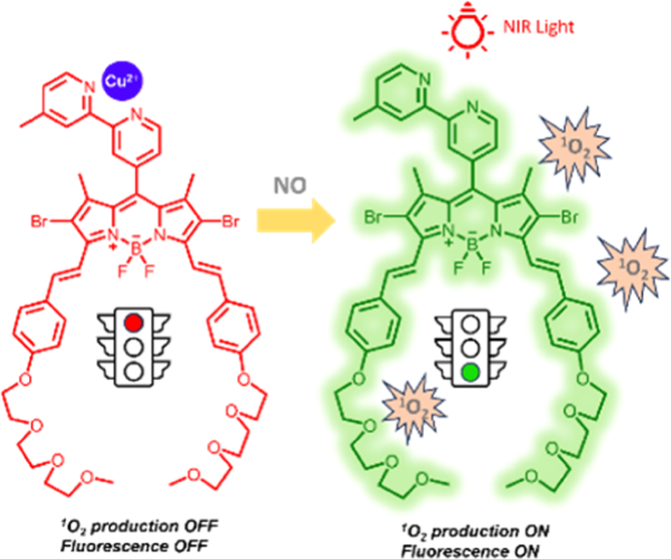

In this work, two BODIPY–bipyridine Cu^2+^ ion complexes for targeted nitric oxide (NO) activatable photodynamic therapy are reported. The design is based on the relatively high concentration of these small gas molecules in the tumor microenvironment. Copper(II) ion complexation to the photosensitizer renders it in the OFF position in terms of fluorescence and reactive oxygen species (ROS) production. The interaction of the Cu^2+^–BODIPY complex with nitric oxide interchanges both fluorescence and therapy mode into the ON state through the detachment of the cation. Therefore, targeting the cancer cells would be expected to be achieved in this way. Moreover, one of the compounds, **AP5**, has increased aqueous solubility due to the polar structure. The designed structures also have near-infrared (IR) absorption ability up to 800 nm aqueous solutions. In addition, through using in vitro cell culture studies with HeLa and RAW264.7 cell lines, we confirmed that **AP5** and **AP6** could be activated in the presence of NO, and cell photocytotoxicity occurred extensively compared with the NO-absent cells. We believe that this work will provide new opportunities for the increased efficacy of the photodynamic treatment of cancer and smart photosensitizer design.

Cancer is known as one of the major health problems with a high mortality rate. Every year, many people are diagnosed with cancer. It was reported recently that in one year, nearly 20 million people were diagnosed with cancer and 10 million people died due to cancer.^1^ The most common treatment methods involve a combination of techniques in which the primary tumor is removed by a surgical procedure, and the patient is further treated with immunotherapy, radiotherapy, or chemotherapy.^2^ Photodynamic therapy (PDT) is complementary and alternative to these traditional treatments and is used to treat both cancerous and noncancerous diseases.^3−6^ Further research opportunities exist in PDT research, and BODIPY-based compounds are frequently used.^7−15^ Activatable photosensitizers (PSs) are one of the main focuses of research to boost efficiency and decrease off-target photosensitization, which could provoke additional complications.^16−18^ To achieve this goal, cancer-related cellular parameters and biomarkers such as low pH,^7,19^ high glutathione concentration,^20−22^ hypoxia,^23^ and enzyme activation^24−28^ (e.g., cathepsin B and caspase) have been considered. One of the rare cellular parameters related to tumorous tissues is nitric oxide (NO).^29,30^ The concentration of NO and the expression of NOSs are determined to be relatively higher in cancer cells compared to normal cells.^31,32^ The high concentration of NO in tumor cells was reported to have both tumor-promoting and -inhibiting effects. Tumor promotion by NO is carried out through the activation of tumor cell proliferation and the expression of angiogenic factors. Although NO could have tumor-inhibiting effects by causing tumor cell death through DNA damage and genetic mutation, tumors could obtain apoptosis resistance and tumor progression can be observed. In the literature, to the best of our knowledge, there is only one study that includes a NO-activatable PS, which is reported by Hu et al., and the mechanism and the PS used are totally different.^30^ They have synthesized an *o*-phenylenediamine-conjugated bis(phenylethynyl)benzene derivative, and photoinduced electron transfer from the amine units to the PS core was observed. Upon treatment with NO, the cleavage of the amine units turned the ^1^O_2_ production on.

BODIPY chromophores are an important class of chromophores due to their superior properties, such as high fluorescence quantum yield and relatively easy functionalization.^7,33−37^ Previously, Costero et al. reported a BODIPY-based chemosensor to detect NO in air and in cells.^38^ When the bipyridine moiety is placed at the *meso* position of the BODIPY core and forms a complex with the Cu^2+^ ion, fluorescence is quenched via forming a weakly emissive charge transfer state. In this research, we used a similar structure and tested the fluorescence, as well as ROS production quenching ability, with near-infrared (IR) absorption and water-soluble characteristics. As far as we are aware, there is no other report where significant quenching of ^1^O_2_/ROS production of a BODIPY-based photosensitizer takes place upon metal complexation. Previous reports suggest that complexation of various metal ions can decrease the singlet oxygen production efficiency due to complexation of paramagnetic copper ions.^39−42^ In our recently published study, we have shown that direct hydrazone substitution to the BODIPY core from the 2-position quenches both fluorescence and singlet oxygen production.^7^ Therefore, we envisaged that quenching or a decrease of both fluorescence and ROS production would also be the case in this design. Based on this assumption, two BODIPY-based compounds (hydrophilic **AP5** and more hydrophobic **AP6**; Figure 1) were synthesized, and their complexes with Cu^2+^ were considered. In the presence of NO, the Cu^2+^ ion in the complex was removed by NO through reduction to Cu^+^, and the fluorescence and ROS production were restored. For in vitro studies, RAW264.7 murine macrophage-like cells and cervical cancer HeLa cell lines were used, and the activation of the PS with NO was accomplished. Prodrugs **AP5** and **AP6** then could be alternative photosensitizer agents for targeted photodynamic therapy, since the activities are expected to be limited outside the tumor microenvironment due to the low concentration of NO. Then, they can be selectively activated in the tumorous region, can produce cytotoxic ROS, and can be used for tumor removal. The activation could also be monitored through fluorescence.

**Figure 1:**
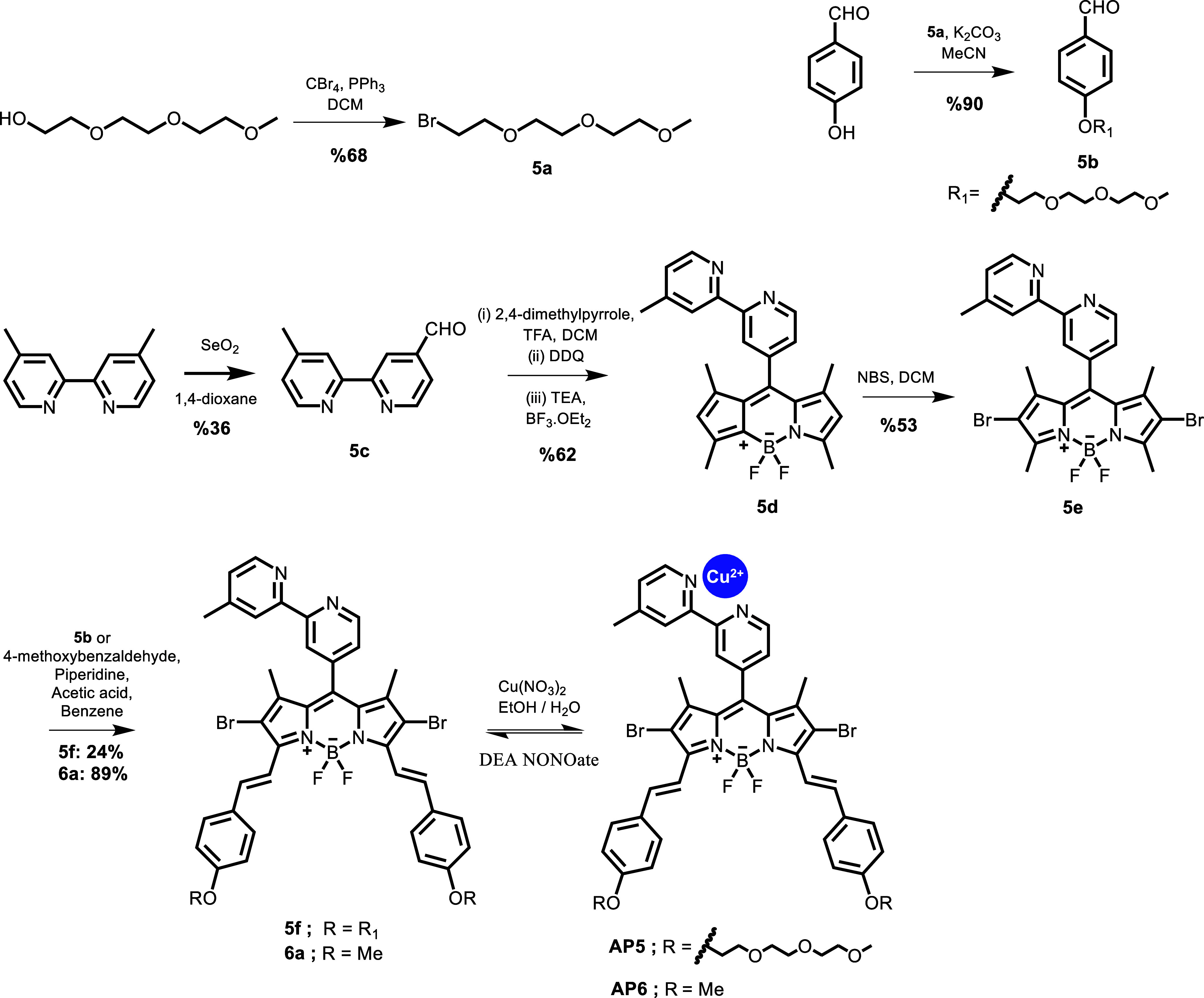
Synthesis scheme for NO-activatable photosensitizers **AP5** and **AP6**.

## Results and Discussion

### Synthesis

Synthesis of the compounds involved seven steps for **AP5** and five steps for **AP6** (Figure 1). For **AP5**, synthesis was started with bromination of triethylene glycol monomethyl ether using tetrabromomethane and triphenylphosphine in DCM to obtain molecule **5a** in 68% yield. Then, using 4-hydroxybenzaldehyde and **5a**, an etherification reaction was performed and **5b** was obtained in 90% yield. Oxidation of one of the methyl units on 4,4′-dimethyl-2,2′-bipyridine was obtained using SeO_2_ in anhydrous 1.4-dioxane, and compound **5c** was obtained in 36% yield.^43^ Then, using **5c** and 2,4-dimethypyrrole, BODIPY compound **5d** was obtained in 62% yield.^38^ Subsequently, dibromination of BODIPY **5d** was achieved to obtain molecule **5e**. By using aldehyde **5b** and BODIPY derivative **5e**, the Knoevenagel condensation reaction was employed to get the molecule **5f** in 24% yield. To obtain compound **6a**, 4-methoxybenzaldeyde and BODIPY derivative **5e** were used, and the yield was recorded as 89%. In the final step, complexation of bipyridine–BODIPY compounds **5f** and **6a** with Cu^2+^ ions was achieved in situ using Cu(NO_3_)_2_ in MeCN to obtain **AP5** and **AP6**, respectively.

### Photophysical Characterization

To look into the behavior of the synthesized structures with light, absorbance and fluorescence spectra were employed. Photophysical characterization of molecules **5f**, **AP5**, **6a**, and **AP6** was carried out in DCM and MeCN (Figures 1 and S1–S4). The maximum absorbance wavelength of the Cu^2+^ complex, **AP5**, was recorded as 677 nm in acetonitrile and slightly shifted to higher wavelengths compared to parent compound **5f**. Their fluorescence spectra were also recorded, and huge differences between **5f** and **AP5** were observed. Although **5f** is fluorescent (λ_fluo,max_ = 698 nm) with a fluorescence quantum yield of 0.13, Cu^2+^ complex **AP5** is nonfluorescent due to the formation of a weakly emissive charge transfer state (Table 1).^38,44,45^

**Figure 2:**
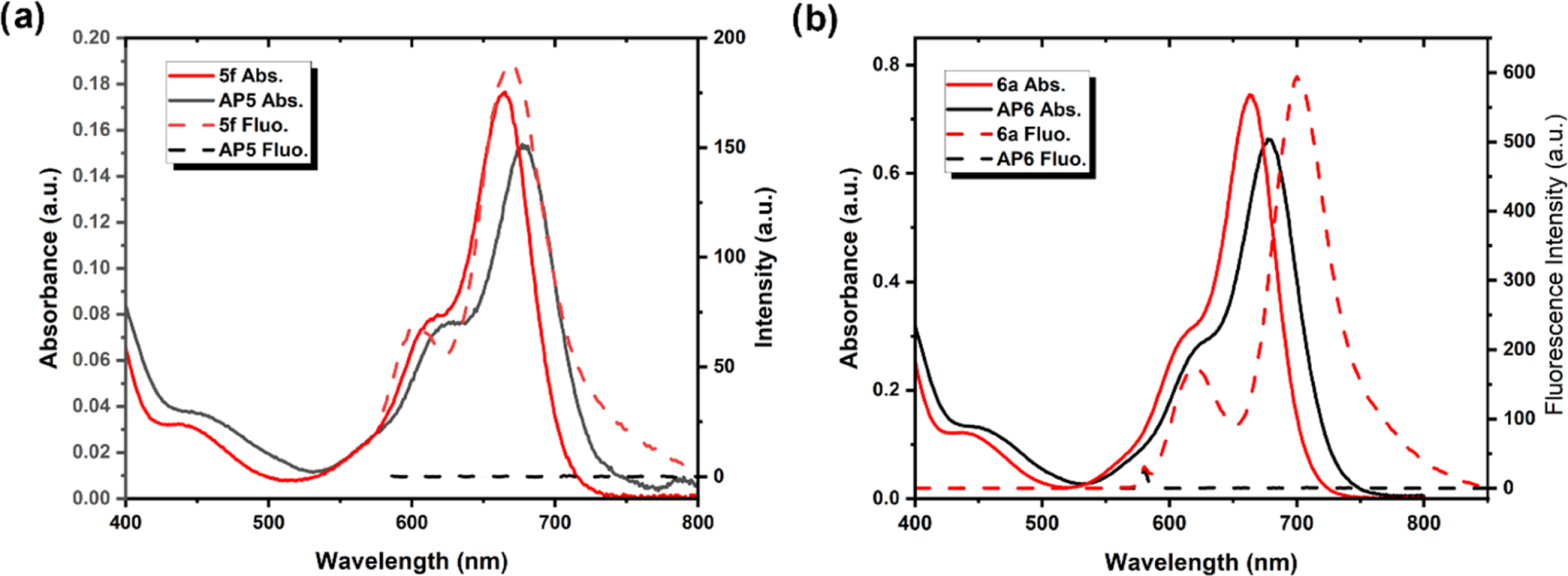
Absorbance and fluorescence spectra of compounds (a) **5f**, **AP5** and (b) **6a**, **AP6** (in MeCN, 1 × 10^–5^ M, for fluorescence excited at 660 nm).

Similar results were observed with the second class of compounds **6a** and **AP6** in terms of absorbance and fluorescence characteristics (Figure 2b). These two structures have higher molar absorptivities compared to **5f** and **AP5**, and compound **6a** has a higher fluorescence quantum yield compared to **5f**, which were calculated as 0.23 and 0.13, respectively (see Table S1 for detailed characterization). A similar compound to **6a** without bromine atoms was also reported before by Qiao et al.^46^ The spectroscopic properties are similar; however, they have observed singlet oxygen formation upon coordination to Ru(II) as expected, and the fluorescence quantum yield was decreased upon coordination. Another work by Padrutt et al. was accomplished using platinum complexes of pyridine–BODIPY. The iodinated compound displays a high phototoxic index in the form of a platinum complex.^47^

**Table 1:** Photophysical Characteristics of the Synthesized Compounds

PS	λ_max,abs_^b^ (nm)	λ_max,fluo_^b^ (nm)	ε_max_^b^ (M^–1^ cm^–1^)	Φ_fluo_^a^^,^^c^	Φ_Δ_^a^^,^^b^
**5f**	665	698	16,000	0.13	0.35
**AP5**	677		13,780	<0.001	0.08
**6a**	663	700	42,000	0.23	0.47
**AP6**	677		37,520	<0.001	0.05

a Fluorescence quantum yields were calculated by using zinc phthalocyanine excited at 650 nm in pyridine as the reference fluorophore (Φ_fluo_ = 0.3),^48^ and for ROS quantum yield, methylene blue was used as the reference compound (Φ_Δ_ = 0.53 in MeCN).^49^

b In MeCN.

c In CHCl_3_.

### Metal Ion Coordination and Decomplexation with Nitric Oxide

**Figure 3:**
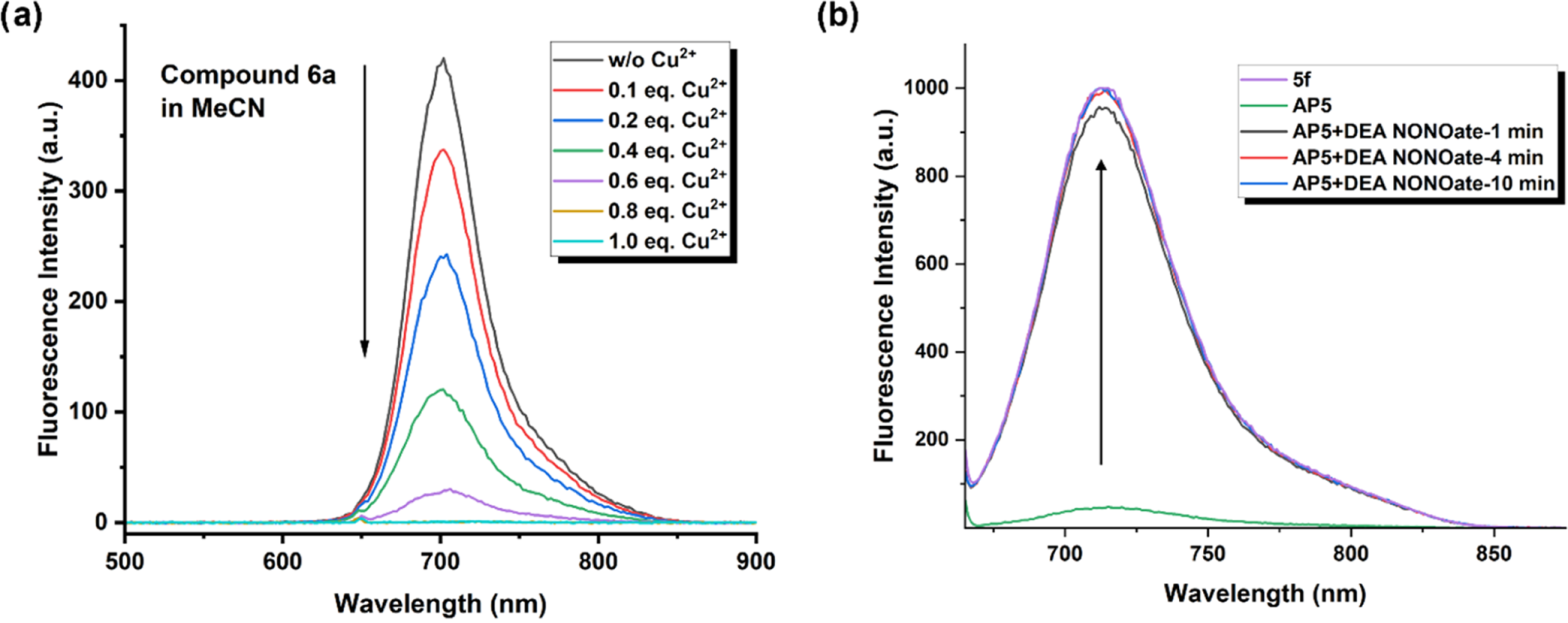
(a) Fluorescence spectra for the titration of compound **6a** with Cu(NO_3_)_2_ in MeCN (1 × 10^–5^ M). (b) Fluorescence spectra of compound **5f**, compound **AP5**, and when DEA NONOate (8 equiv, dissolved in PBS) is added to compound **AP5** (1 × 10^–5^ M, MeCN).

To analyze the selectivity of molecules **5f** and **6a** toward Zn^2+^ and Cu^2+^ ions, metal titration experiments were carried out. Upon the addition of gradually increasing concentrations of metal ions, the changes in the absorbance and fluorescence spectra were evaluated. Upon addition of the metal ions to compound **5f**, a small bathochromic shift in absorbance and a huge decrease in the intensity of fluorescence were recorded (Figures S5 and S6, in MeCN). It was observed that Zn^2+^ ions do not effectively quench the fluorescence; however, with Cu^2+^ ions, complete quenching occurred with 0.8 equiv of metal addition. This observation is probably due to the formation of a more appropriate host–guest complex with Cu^2+^ ions. In the experiments with copper ions, similar results were obtained with **5f** and **6a** (Figures 3a and S6). The quenching of the fluorescence was explained as the formation of a charge transfer state (vide supra). Therefore, further experiments were performed using Cu^2+^ ions to fulfill the complete quenching of both fluorescence and ROS production.

To determine the effect of nitric oxide (NO) in the removal of Cu^2+^ ions from the bipyridine complex in **AP5** and **AP6**, DEA NONOate was used as a NO source. After the removal of the metal ion, corresponding Cu-free host compounds **5f** and **6a** were expected to be reformed. After addition of DEA NONOate (8 equiv, in MeCN) to the solutions of **AP5** or **AP6**, an imminent and strong increase in fluorescence and a slight shift in the absorbance peak to lower wavelengths were observed due to the reformation of ROS-producing compounds **5f** and **6a** (Figures 3b and S10–S11). Therefore, it was determined that nitric oxide could detach Cu^2+^ ions from bipyridine–BODIPY units effectively by reducing it to Cu^+^ ions, since this ion is not a favorable guest for the bipyridine ligand.

As control experiments, we have performed if biological reductants such as cysteine and glutathione have any effect on compounds **AP5**–**6**. After the treatment of the compounds with different equivalents of these compounds, we observed that while cysteine has no effect, glutathione partially restores compounds **AP5**–**6** to active compounds **5f** and **6a**, respectively (Figures S19 and S20). The effect is minimum with **AP5** compared to **AP6**. After 10 equiv of GSH, only half of the fluorescence is restored for **AP5**. The partial activation with GSH indicates that compounds **AP5**–**6** can also be activated partially with this molecule. The increased level of this compound in cancer cells is reported by several studies;^50^ therefore, dual activation with GSH and NO may also be possible.

### Blocking and Unblocking of Reactive Oxygen Species Production

To analyze the ROS production capacities of **5f**, **AP5**, **6a**, and **AP6**, chemical indirect methods were evaluated. 1,3-Diphenylisobenzofuran (DPBF) was used as a reactive oxygen species scavenger, and the decrease in the absorbance of DPBF was quantified as ROS was produced. A light-emitting diode (LED) array as the illumination source was used to expose molecules to specific wavelengths of light (660 nm, 0.03 W/cm^2^). To test the activity of molecules in both organic and aqueous solvent systems, MeCN, phosphate-buffered saline (PBS) buffer, and DMSO–PBS buffer systems were used.

**Figure 4:**
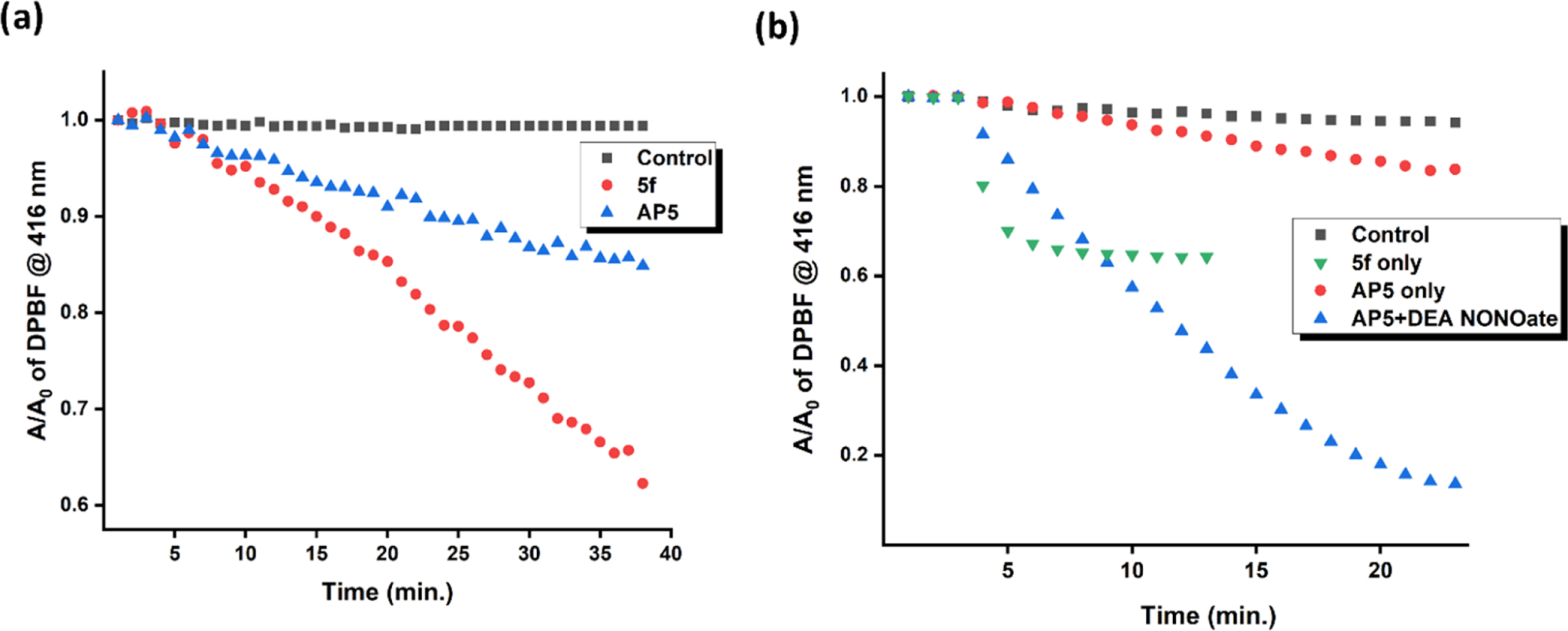
(a) ROS production experiments by tracking the absorbance of the ROS scavenger DPBF in the presence of compound **5f**, **AP5**, and the control experiment without PS (in DMSO/PBS buffer, 4:1); (b) without PS (control), with **AP5**, and with **AP5** + DEA NONOate (8 equiv, in DMSO/PBS buffer, 1:9). In the first 3 min, experiments were performed in the dark (660 nm LED array, 0.03 W/cm^2^).

In the experiments with **5f** and **AP5**, a significant difference between the two was observed (in MeCN or DMSO/PBS buffer (4:1), Figures 4a, S12, and S13). In the DMSO/PBS buffer (4:1), the decrease in the absorbance of DPBF is 15% for **AP5**, while it is 40% for PS **5f** (Figure 4a). This shows the efficient hampering of ROS production upon coordination with the Cu^2+^ ion.

To analyze the unblocking of the ROS production ability of **AP5**, DEA NONOate was used as a NO source. Upon interaction with NO, the formation of **5f** was expected, which is an efficient ROS producer. DEA NONOate was then added to the **AP5**-containing solutions (1 × 10^–5^ M; DMSO/PBS buffer, 4/1 or 1/9), and ROS formation experiments were performed (Figures 4b and S16 and S17). The two triethylene glycol units in **AP5** allowed solubilization in a highly polar environment. In these experiments, we have observed that the unblocking of the ROS production was successfully achieved. When only **AP5** exists, there is minimal change in DPBF absorption; on the other hand, when the solution is treated with NO, the rate of decrease rises sharply, indicating efficient ROS production.

After the addition of compound **AP5**, experiments with **AP6** were evaluated. Likewise, significant differences between **AP6** and **6a** were observed in ROS production capabilities (in both MeCN or DMSO/PBS buffer mixture), where **6a** has a high ROS production capacity (Figures S14 and S15). Next, NO activation experiments were performed, and successful results were attained (Figure S17). In total, 30 min of irradiation with a 660 nm LED array, with sole **AP6**, a minimum decrease in the absorbance of DPBF (ca. 9%) was observed. Conversely, when DEA NONOate was added to **AP6** and irradiated, a huge change (73%) was detected (DMSO/PBS buffer, 4:1, Figure S18).

### In Vitro Studies

The activities of designed molecules **5f** (active), **AP5** (inactive), **6a** (active), and **AP6** (inactive) were assessed through in vitro studies. The highly aggressive cancer cell line HeLa and macrophage-like cells RAW264.7, originating from the Abelson leukemia virus transformed mice cell line known for its inherent nitric oxide production, were utilized as target models.^51^ The cytotoxicity induced by the active molecules was assessed both under dark and under irradiation. For HeLa cells, the activation of molecules **AP5** and **AP6** was induced through the administration of an external nitric oxide source using a chemical reagent, namely, DEA NONOate.^52^ However, this procedure was not conducted for the RAW264.7 cell line, given that macrophage cells inherently possess a high concentration of nitric oxide.^30,38^

**Figure 5:**
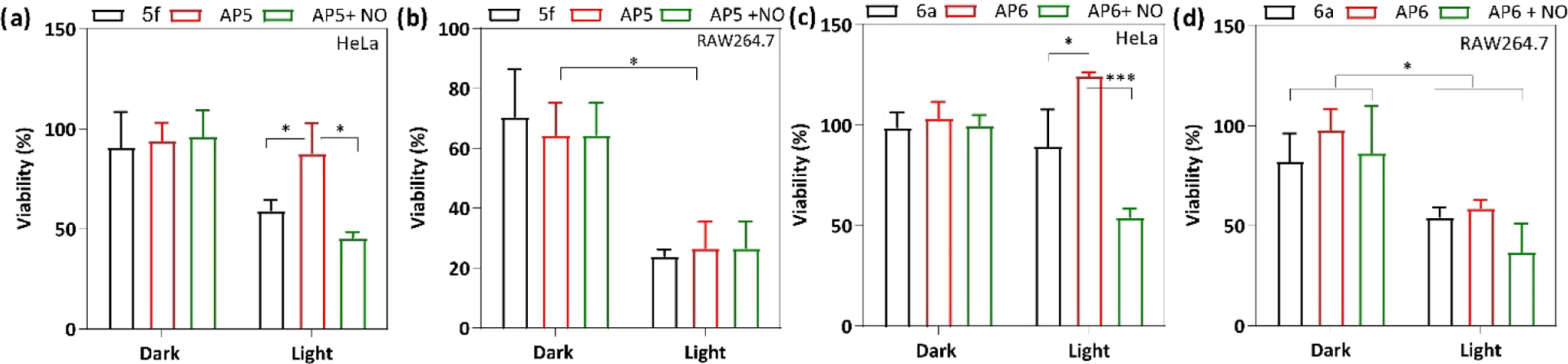
(a) Viability of HeLa cells incubated with **5f**, **AP5**, and **AP5** + DEA NONOate for 24 h and exposed to a 660 nm LED array (0.03 W/cm^2^) for 1 h (1 μM each of the PSs and 5 mM DEA NONOate where appropriate). (b) Viability of RAW264.7 cells under the same conditions as (a) in the presence of **5f** and **AP5** (with and without an external NO source). (c) The viability of HeLa cells incubated with **6a**, **AP6**, and **AP6** + DEA NONOate in the same conditions as (a). (d) Viability of RAW264.7 cells under the same conditions as (a) in the presence of **6a** and **AP6** (with and without an external NO source).

First, unblocked photosensitizers **5f** and **6a** were compared with **AP5** and **AP6** in both cell lines. Before the activity assessment was begun, the internalization profile of the synthesized agents was monitored. A significant amount of the molecules internalized within 4 h, and the process was completed after 24 h. This 24 h period was used as the incubation time for subsequent experiments. Significant differences between **5f**-**AP5** and **6a**-**AP6** sets were observed, indicating that in the Cu^2+^-complexed structures **AP5**–**6**, ROS production abilities were suspended effectively (Figure 5, 1 μM, 660 nm LED array, 0.03 W/cm^2^). Therefore, they do not affect viability as much as unblocked molecules **5f** and **6a**. The addition of nitric oxide (NO) has been found to be significant for unlocking the photosensitizer activity in HeLa cells. However, because RAW264.7 cells inherently contain NO, the external addition of NO only slightly improved the cytotoxicity generated from blocked agent **AP5**–**6** (Figure 5b–d).

**Figure 6:**
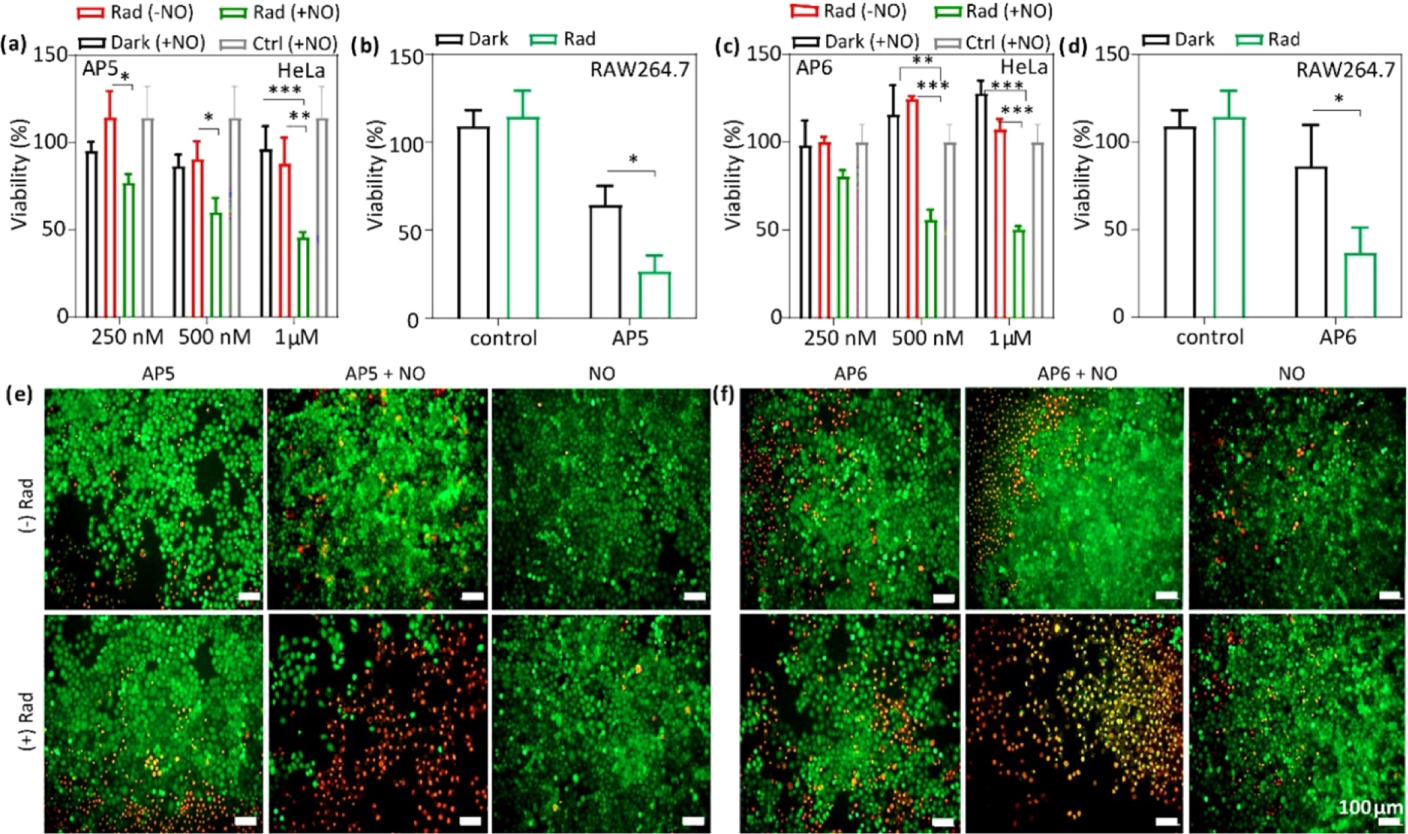
(a–d) Viabilities of the HeLa cell line and RAW264.7 were assessed under varying concentrations of **AP5** and **AP6** under dark and irradiation in the absence/presence of NO. (e) The live/dead assay microscopy images are presented for **AP5**, control group (NO-treated HeLa cells), along with images of the group incubated with **AP5**, and the group incubated with **AP5** + DEA NONOate. (f) For the experiments with **AP6**: the control group (NO-treated HeLa cells) is presented, the group incubated with **AP6**, and the group incubated with **AP6** + DEA NONOate (cells were incubated with agents for 24 h and exposed to a 660 nm LED light (0.03 W/cm^2^) for 1 h; the microscopy images show the experiment group of HeLa cells tested with 1 μM). Calcein/PI was used as the staining agent. Both dark and irradiated samples were presented.

Afterward, HeLa cells were subjected to **AP5** and **AP6** individually and treated with a nitric oxide source for activation. Then, the photocytotoxicities of **AP5** or **AP6** in the absence/presence of NO on cancer cells were detected successfully. For the NO-activated PSs, EC_50_ values for each of them were determined as 1 μM (Figure 6a,c). At 250 nM, a comparable profile was observed between two photosensitizers, but for both agents, the effective cytotoxicity concentration was identified as 1 μM. NO-activated **AP5** has been identified as slightly more efficient, initiating the death of approximately 65% of the cell population, whereas NO-activated **AP6**, at the same concentration, induces approximately 45% cell death. Importantly, in the absence of nitric oxide activation or in the only NO-treated groups, no notable cytotoxicity was observed. There are some reports in the literature about the ROS production ability of the Cu^+^ and Cu^2+^ ions.^53,54^ Therefore, as a control group, only Cu^2+^-added HeLa cell lines were also studied, and it was observed that with 1 μM concentration, copper ions have minimal effect on photocytotoxicity (Figure S23). These observations signify the effectiveness of nitric-oxide-dependent activation for the designed prodrugs. The reduction in cell viability upon activation of blocked photosensitizers was also assessed through fluorescence microscopy studies. These images revealed a similar trend with quantitative findings; apoptotic cell morphology and a decrease in cellular viability caused by activated agents were illustrated (Figures 6e–j and S24). In the review article by Fukumura, it has been stated that in many tumors, the expression levels and activities of the different NOSs (nitric oxide synthases) are higher compared with corresponding normal tissues.^29^ Also, Thomsen and Miles specified that the expression of NOs in human cancers such as the breast, stomach, ovary, cervix, and central nervous system has been reported.^32^ Since it is difficult to obtain the exact tumor microenvironment with in vitro studies, we have not observed NO activity in HeLa cancer cells without addition of an external NO source. However, RAW264.7 macrophage cell lines provide a decent model, since the appearance of NO in the cancerous environment is attributed to taking part in intratumoral macrophages.^32^

**Figure 7:**
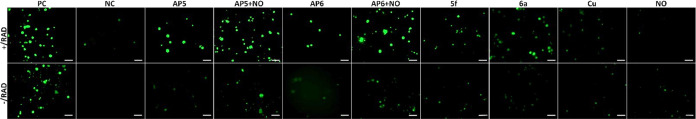
Fluorescence microscopy images of the DCFH-DA ROS assay of the HeLa cells in the presence of inactive agents **AP5** and **AP6**, active agents **5f** and **6a**, NO-activated groups **AP5** + NO and **AP6** + NO, and only NO and only Cu^2+^-treated groups. In this study, ROS production is used to induce cytotoxicity in cancer cells. The formation of activated ROS was investigated by using a reactive oxygen species (ROS) assay. Cells were treated with 0.5 μM blocked and unblocked compounds in the presence or absence of NO and 660 nm LED light. PC: Positive control.

Nitric oxide is produced in RAW264.7 macrophage cells in high concentrations in response to stress conditions.^55^ Therefore, AP prodrugs are expected to be activated in situ when treated with these cell lines without any external NO administration. Upon treatment of **AP5** and **AP6** in these cell lines, we observed effective activation of the prodrugs, and powerful photocytotoxicity was observed (Figure 6b,d). **AP5** induced approximately 77% cell death, while **AP6** led to 63% cell death. It is of note that dark toxicities were observed to some extent with these cell lines. NO inhibition control experiments were also performed using quercetin (Q) molecules^56^ (Figure S27). An increase of more than 30% in viability was observed when RAW264.7 cells were preincubated with Q, compared with the group treated with **AP5** alone. Despite the significant improvement in viability, a notable rate of cell death was observed in the **AP5 + Q** group. Further experiments with fluorescence spectroscopy indicated that quercetin could partially decomplex Cu^2+^ from **AP5**, converting it into PDT-active molecule **5f** (Figures S21 and S22). It is believed that this could have contributed to the increased cell toxicity observed in the **AP5 + Q** group. ROS generation was illustrated using the DCFH-DA ROS assay by the development of fluorescent signals and compared to a ROS-generating positive control (*tert*-butyl hydroperoxide, *t*-BHP). *t*-BHP led to fluorescent signal generation, which was considered adequate to compare the ROS generation of the activatable agent. Consistent with the cytotoxicity results, the fully activated groups showed higher ROS generation, while the control groups did not exhibit significant signals (Figure 7). These results indicate that the observed activity resulted from the ROS formation upon NO activation. Even at much lower concentrations (1 μM), the synthesized photosensitizers showed comparable ROS generation to the positive control group (100 μM).

To conclude, upon administration of nitric oxide, levels of induced cytotoxicity for **AP5** + NO and **AP6** + NO were found to be statistically similar to those of **5f** and **6a**. Both HeLa and RAW264.7 cells were employed for this comparative study, and similar trends were observed. Therefore, **AP5** and **AP6** prodrugs can be used selectively to target cancer cells, and PDT treatment can be carried out while avoiding off-target effects. Furthermore, it can provide additional applications, such as extending its use to immunogenic diseases.

## Conclusions

In this work, two BODIPY derivatives, **AP5** and **AP6**, were designed as activatable photosensitizers for activation in nitric oxide-rich cancer cells. After the synthesis of the smart PSs with good yield, photophysical characterization of the target and precursor compounds, activation through nitric oxide, and blocking/unblocking of the ROS production experiments were carried out. Activatable PSs, **AP5** and **AP6**, absorb light in the near-infrared region (up to 800 nm in aqueous media), which is regarded as a critical parameter for enhanced light penetration through the body. One of the compounds, **AP5**, has increased aqueous solubility due to the polar structure. When BODIPY derivatives are substituted with bromine/iodine atoms, their core ROS production ability is induced. However, in our work, fluorescence and ROS production efficiencies were restricted upon metal ion binding to the bipyridine unit at the *meso* position of the BODIPY core due to formation of a charge transfer state. Therefore, passage to the triplet state does not occur efficiently. But then, in the presence of nitric oxide, Cu^2+^ ions were reduced to the Cu^+^ ions and removed from the complex, since it is not a suitable guest for the bipyridine unit. Consequently, the ROS production ability in the cancerous tissues is expected to be turned on. The activation of the prodrug in the other parts of the body is expected to be diminished due to the low concentration of NO in normal cells. Therefore, targeting of the cancer cells would be achieved in this way. Moreover, cell culture studies using HeLa and RAW264.7 confirmed that **AP5** and **AP6** could be activated in the presence of nitric oxide, and cell photocytotoxicity occurred extensively compared with the NO-absent cells. We believe this study will provide new opportunities for the increased efficacy of the photodynamic treatment of cancer.

## Methods

### General

All chemicals and solvents purchased from Aldrich, Acros, or TCI were used without further purification unless otherwise stated. ^1^H NMR and ^13^C NMR spectra were recorded using a Bruker DPX-400 in CDCl_3_ with TMS as the internal reference. Column chromatography of all products was performed using Merck Silica Gel 60 or Silicycle (particle size: 0.040–0.063 mm, 230–400 mesh ASTM). Reactions were monitored by thin-layer chromatography using fluorescent-coated aluminum sheets. Absorption spectrometry in solution was performed using an Agilent Cary 60 spectrophotometer. Steady-state fluorescence measurements were conducted using an Agilent Eclipse spectrofluorometer. Solvents used for spectroscopy experiments were spectrophotometric grade. Fluorescence quantum yields were calculated by using the method in the literature^57^ using the reference dye zinc phthalocyanine.^48^ For ROS quantum yield, methylene blue was used as a reference compound.^49^

### Synthesis

#### Compound **5a**(58)

Triethylene glycol monomethyl ether (12.18 mmol, 2 g, 1.95 mL, 1 equiv) and carbon tetrabromide (13.39 mmol, 4.44 g, 1.1 equiv) were dissolved in 30 mL of dry dichloromethane. The solution was cooled in an ice bath and stirred. Then, triphenylphosphine (14.62 mmol, 3.83 g, 1.2 equiv) was added to this solution in fractions for 15 min. The solution was stirred for an additional 1 h at room temperature. The progress of the reaction was monitored through thin-layer chromatography (TLC), and the reaction was terminated after 1 h. The crude product was purified by silica gel column chromatography using ethyl acetate/hexane (1:1) as the eluent. The fraction containing **5a** was collected, and then the solvent was evaporated under reduced pressure.1.89 g (8.32 mmol) of the pure product was obtained in 68% yield and as a yellow liquid. ^1^H NMR (400 MHz, Chloroform-*d*) δ 3.81 (t, *J* = 6.3 Hz, 2H), 3.71–3.63 (m, 6H), 3.58–3.53 (m, 2H), 3.47 (t, *J* = 6.4 Hz, 2H), 3.38 (s, 3H). ^13^C NMR (101 MHz, CDCl_3_) δ: 71.93, 71.21, 70.61, 70.60, 70.53, 59.05, 30.30. High-resolution mass spectrometry (HRMS) (TOF-ESI): *m*/*z* C_7_H_15_BrO_3_ calcd as 227.0277 [M + H]^+^; found as 227.02739.

#### Compound **5b**

Modified from literature.^59^ Compound **5a** (4.4 mmol, 1 g, 1.2 equiv) and 4-hydroxybenzaldehyde (3.67 mmol, 0.45 g) were dissolved in acetone, and finally, potassium carbonate (K_2_CO_3_) (14.67 mmol, 2.028 g, 4 equiv) was added to the solution. The solution was refluxed at 66 °C for 24 h. The acetone in the reaction mixture was evaporated under reduced pressure. Then, water (100 mL) and ethyl acetate (100 mL) were added to the reaction mixture for solvent extraction. The organic layer was collected and dried with sodium sulfate, and ethyl acetate was evaporated under reduced pressure. The crude product was purified by silica gel column chromatography using ethyl acetate/hexane (4:1) as the eluent. Product **5b** was obtained in 90% yield and as a yellow liquid (0.8817 g., 3.96 mmol). ^1^H NMR (400 MHz, Chloroform-*d*) δ 9.88 (s, 1H), 7.92–7.77 (m, 2H), 7.05–6.99 (m, 2H), 4.25–4.17 (m, 2H), 3.93–3.85 (m, 2H), 3.77–3.71 (m, 2H), 3.71–3.62 (m, 4H), 3.60–3.48 (m, 2H), 3.37 (s, 3H). ^13^C NMR (101 MHz, CDCl 3) δ 190.82, 163.87, 131.95, 130.06, 114.89, 71.93, 70.92, 70.67, 70.60, 69.49, 67.77, 59.05. HRMS (TOF-ESI): *m*/*z* C_14_H_20_O_5_ calcd as 269.13835 [M + H]^+^; found as 269.13789.

#### Compound **5c**

This compound was synthesized according to the literature.^43^ 4,4-Dimethyl-2,2-bipyridine (5.43 mmol, 1 g, 1 equiv) was added in small fractions to 40 mL of dry 1,4-dioxane and stirred. The solution was degassed with N_2_ for 15 min, and then selenium dioxide (5.997 mmol, 0.6632 g, 1.1 equiv) was added to the solution. The reaction mixture was refluxed under a nitrogen atmosphere for 24 h. The reaction mixture was washed with warm 1,4-dioxane three times and filtered. 1,4-Dioxane was removed under vacuum. The obtained precipitate was dissolved in warm ethyl acetate, filtered, and washed with warm ethyl acetate. To remove carboxylic acid from the reaction mixture, the organic phase was washed with 250 mL of 1 M Na_2_CO_3_ solution. The organic layer was dried, and ethyl acetate was evaporated in vacuum. The crude product was purified by silica gel column chromatography using ethyl acetate–hexane (2:3) as the eluent. The fraction containing **5c** was collected, and then the solvent was evaporated under reduced pressure (36% reaction yield, 1.95 mmol, 385 mg). ^1^H NMR (400 MHz, Chloroform-*d*) δ 10.19 (s, 1H), 8.90 (d, *J* = 4.9 Hz, 1H), 8.86–8.81 (m, 1H), 8.58 (d, *J* = 4.9 Hz, 1H), 8.28 (s, 1H), 7.77–7.67 (m, 1H), 7.23–7.16 (m, 1H), 2.47 (s, 3H). HRMS (TOF-ESI): *m*/*z* C_12_H_10_N_2_O calcd as 199.08659 [M + H]^+^; found as 199.08666.

#### Compound **5d**

Compound **5d** was synthesized according to the literature.^38^ To synthesize **5d**, dichloromethane was degassed with N_2_ for 15 min, and then molecule **5c** (1.841 mmol, 0.365 g, 1 equiv), 2,4-dimethylpyrrole (3.682 mmol, 350 mg, 2 equiv), and three drops of trifluoroacetic acid (TFA) were added. The reaction mixture was stirred for 24 h under N_2_. Then, 2,3-dichloro-5,6-dicyano-1,4-benzoquinone (DDQ, 1.841 mmol, 0.417 mg, 1 equiv) was added. This mixture was stirred for 1.5 h, and then triethylamine (TEA) (2.2 mL) was added. The reaction mixture was stirred for an additional 30 min, and then boron trifluoride etherate (BF_3_·OEt_2_) (2.2 mL) was added. This reaction mixture was stirred for 1 h. Then, 150 mL of water and 150 mL of DCM were added for solvent extraction. The organic layer was collected and dried with sodium sulfate, and DCM was evaporated under reduced pressure. The crude product was purified by silica gel column chromatography using ethyl acetate–hexane (4:1) as the eluent. The fraction containing **5d** was collected. The product was obtained in 62% yield. ^1^H NMR (400 MHz, Chloroform-*d*) δ 8.82 (d, *J* = 4.9 Hz, 1H), 8.52 (d, *J* = 5.0 Hz, 1H), 8.48–8.43 (m, 1H), 8.31 (s, 1H), 7.33–7.28 (m, 1H), 7.17 (d, *J* = 4.3 Hz, 1H), 5.99 (s, 2H), 2.56 (s, 6H), 2.47 (s, 3H), 1.46 (s, 6H). HRMS (TOF-ESI): *m*/*z* C_24_H_23_BF_2_N_4_ calcd as 417.20566 [M + H]^+^; found as 417.21340.

#### Compound **5e**

To synthesize **5e**, *N*-bromosuccinimide (0.56 mmol, 99.79 mg, 2.4 equiv) was added to 32 mL of 1:1 dichloromethane-dimethylformamide and stirred at room temperature, without light exposure, for 1 h. The reaction was terminated by monitoring the reaction mixture with TLC. After the reaction was completed, saturated Na_2_S_2_O_3_ (50 mL) solution was added to the mixture, and it was diluted with DCM. The aqueous phase was extracted with DCM twice, and then the collected organic phase was washed with brine and dried with Mg_2_SO_4_. DCM was removed under vacuum. The crude product was purified by silica gel column chromatography, using 4% methanol in DCM as the eluent. The fraction containing **5e** was collected and the product was obtained in 53% yield (0.124 mmol, 70.4 mg). ^1^H NMR (400 MHz, Chloroform-*d*) δ 8.79 (s, 1H), 8.42 (d, *J* = 10.8 Hz, 2H), 8.26 (s, 1H), 7.26–7.17 (m, 1H), 7.11 (s, 1H), 2.55 (s, 6H), 2.40 (s, 3H), 1.40 (s, 6H). ^13^C NMR (101 MHz, CDCl_3_) δ: 157.6, 154.8, 154.5, 150.1, 149.3, 148.4, 143.8, 140.2, 138.5, 129.5, 125.5, 122.6, 122.0, 120.6, 112.3, 21.3, 14.3, 13.8. HRMS (TOF-ESI): *m*/*z* C_24_H_21_BBr_2_F_2_N_4_ calcd as 575.02464 [M + H]^+^; found as 575.02504.

#### Compound **5f**

Molecule **5e** (0.1219 mmol, 70 mg, 1 equiv) was dissolved in 10 mL of benzene, and **5b** (0.3658 mmol, 98.15 mg, 3 equiv) was also added to the solution. After adding piperidine (219 μL) and acetic acid (219 μL) to the solution, the reaction mixture was refluxed at 100 °C. The progress of the reaction was monitored through TLC, and when the product formation was completed, the reaction was terminated. After the reaction was terminated, it was extracted with 150 mL of water and 50 mL of DCM (the organic layer was washed with water three times). The organic layer was collected and dried with sodium sulfate, and DCM was evaporated in vacuum. To purify the product, preparative TLC was used (3% methanol in DCM), and molecule **5f** was obtained with a reaction yield of 23% (31 mg). ^1^H NMR (400 MHz, CDCl_3_) δ 8.79 (d, *J* = 4.9 Hz, 1H), 8.52–8.39 (m, 2H), 8.27 (s, 1H), 8.06 (d, *J* = 16.6 Hz, 2H), 7.61–7.47 (m, 6H), 7.32–7.23 (m, 1H), 7.12 (d, *J* = 4.4 Hz, 1H), 6.89 (d, *J* = 8.7 Hz, 4H), 4.12 (t, *J* = 4.8 Hz, 4H), 3.83 (t, *J* = 4.8 Hz, 4H), 3.72–3.67 (m, 4H), 3.66–3.62 (m, 4H), 3.62–3.56 (m, 4H), 3.53–3.43 (m, 4H), 3.32 (s, 6H), 2.41 (s, 3H), 1.43 (s, 6H). ^13^C NMR (101 MHz, CDCl_3_) δ: 176.55, 159.12, 156.46, 153.63, 148.98, 148.28, 147.96, 147.37, 143.47, 139.35, 138.35, 130.94, 130.08, 128.80, 128.37, 124.39, 122.33, 120.99, 120.31, 114.98, 113.98, 113.87, 109.58, 70.93, 69.88, 69.67, 69.58, 68.67, 66.54, 58.04, 28.68, 13.26. HRMS (TOF-ESI): *m*/*z* C_52_H_57_BBr_2_F_2_N_4_NaO_8_^+^ calcd as 1097.24760 [M + Na]^+^; found as 1097.24545.

#### Compound **6a**

To synthesize molecule **6a**, **5e** (0.084 mmol, 48.5 mg, 1 equiv) was dissolved in 15 mL of benzene, and 4-methoxybenzaldehyde (0.25 mmol, 34.5 mg, 3 equiv) was also added to the solution. After adding piperidine (300 μL) and acetic acid (300 μL) to the solution, the reaction mixture was refluxed at 100 °C using a Dean–Stark apparatus. Progress of the reaction was monitored through TLC, and when the product formation was completed, the reaction was terminated. After the reaction was terminated, it was extracted with 150 mL of water and 50 mL of DCM (the organic layer was washed with water three times). The organic layer was collected and dried with sodium sulfate, and DCM was evaporated in vacuum. To purify the product, 3% methanol in a DCM solvent system was used in column chromatography, and **6a** was obtained with a reaction yield of 89% (62.7 mg). ^1^H NMR (400 MHz, CDCl_3_) δ 8.80–8.73 (m, 1H), 8.51–8.45 (m, 2H), 8.29–8.24 (m, 1H), 8.06 (d, *J* = 16.6 Hz, 2H), 7.61–7.48 (m, 6H), 7.27–7.23 (m, 1H), 7.17–7.08 (m, 1H), 6.85 (d, *J* = 8.8 Hz, 4H), 3.78 (s, 6H),2.41 (s, 3H), 1.41 (s, 6H).^13^C NMR (101 MHz, CDCl_3_) δ 160.91, 157.50, 154.70, 150.00, 149.34, 148.97, 148.37, 144.51, 140.38, 139.33,134.93, 131.12, 129.72, 129.42, 125.41, 123.36, 122.00, 121.34, 116.01, 114.35, 110.58, 77.35, 77.23, 77.03,76.71, 55.42, 21.26, 14.14. HRMS (TOF-ESI): *m*/*z* C_40_H_34_BBr_2_F_2_N_4_O_2_^+^ calcd as 811.10837 [M + H]^+^; found as 811.10803.

#### Compounds **AP5** and **AP6**

In the synthesis of **AP5** and **AP6**, 1 equiv of copper(II) nitrate trihydrate (Cu(NO_3_)_2_·3 H_2_O) was added to the solution of **5f** or **6a** to obtain in situ formation of copper complexation. **AP5**: HRMS (TOF-ESI): *m*/*z* C_52_H_57_BBr_2_CuF_2_N_4_O_8_ (M + Cu)^+^ calcd as *m*/*z* 1137.18743, found as 1137.20014, Δ = 11.18 ppm. **AP6**: HRMS (TOF-ESI): *m*/*z* C_40_H_33_BBr_2_CuF_2_N_4_O_2_, (M + Cu)^+^ calcd as 873.03014, found as 873.04003. Δ = 11.33 ppm.

## Supporting Information Available

Additional data including experimental procedures; photophysical characterization; NMR spectra; mass spectra; HPLC traces; and data for biological studies (PDF)
