# Supplementary material for: Nitric Oxide Activatable Photodynamic Therapy Agents Based on BODIPY–Copper Complexes
Source: ACS Pharmacol Transl Sci. 2024 Dec 10;8(3):679–89. doi: 10.1021/acsptsci.4c00428 (PMC11915034; doi:10.1021/acsptsci.4c00428)
Supplement: Supplementary file 1 — pt4c00428_si_001.pdf [file pt4c00428_si_001.pdf]

SUPPORTING INFORMATION

Nitric Oxide Activatable Photodynamic Therapy Agents Based on BODIPY-Copper Complexes

Huriye Ilhan[1,4], Merve Şeker[2], Gülcihan Gülseren[2], Melike Ebrar Bakırcı[2], Ayşe İlayda Boyacı[3], Yusuf Cakmak[4]

[1]Department of Biotechnology, Graduate School of Natural & Applied Sciences, Konya Food and Agriculture University, 42080 Konya, Turkey
[2]Department of Molecular Biology and Genetics, Konya Food and Agriculture University, 42080 Konya, Turkey
[3]Department of Molecular Biology and Genetics, Necmettin Erbakan University, 42090 Konya, Turkey
[4]Department of Metallurgical and Materials Engineering & Science and Technology Research and Application Center (BITAM), Necmettin Erbakan University, 42090 Konya, Turkey

E-mail: yusuf.cakmak@erbakan.edu.tr

Contents

1 General Experimental Details 2
2 Additional Photophysical Characterization Data 2
2.1 Fluorescence Quantum Yield and Singlet Oxygen Quantum Yield Determination 3
2.2 Additional Absorbance and Fluorescence Spectra 3
2.3 Metal Titration Experiments: 5
2.4 Nitric oxide addition experiments: 7
2.5 Reactive Oxygen Species (ROS) Determination Experiments: 8
2.6 ROS Determination Experiments upon NO activation: 11
2.7 Interaction of AP5 and AP6 with Cysteine and Glutathione: 13
2.8 Interaction of AP5 and AP6 with Quercetin 14
3 Cell Culture Experiments and Fluorescence Microscopy Imaging 15
3.1 Live/Dead Assay: 16
3.2 ROS Assay : 16
3.3 Internalization: 17
3.4 NO Inhibition Control Experiments: 19
4 ^{1}H and ^{13}C NMR Data 20
5 High Resolution Mass Spectrometer Data 28
6 References 34

1 General Experimental Details

All chemicals and solvents purchased from Aldrich, Acros or TCI were used without further purification unless otherwise stated. ^{1}H NMR and ^{13}C NMR spectra were recorded using a Bruker DPX-400 in $CDCl_3$ with TMS as internal reference. Column chromatography of all products was performed using Merck Silica Gel 60 or Silicycle (particle size: 0.040–0.063 mm, 230–400 mesh ASTM). Reactions were monitored by thin layer chromatography using fluorescent coated aluminum sheets. Absorption spectrometry in solution was performed using Agilent Cary 60 spectrophotometer. Steady state fluorescence measurements were conducted using an Agilent Eclipse spectrofluorometer. Solvents used for spectroscopy experiments were spectrophotometric grade. Fluorescence quantum yields were calculated by using the method in the literature[1] using the reference dye Zinc phthalocyanine was used[2]. For singlet oxygen quantum yield methylene blue was used as reference compounds[3]. All compounds that are used in *in vitro* studies are >95% pure by HPLC analysis.

2 Additional Photophysical Characterization Data

Table S1. Molar absorptivity, fluorescence quantum yield (using reference molecule zinc phthalocyanine) and singlet oxygen quantum yield (using reference molecule methylene blue) values for molecules **AP5**, **5f**, **AP6** and **6a**.

PS	λ_{max}, abs. (nm)	λ_{max}, fluo. (nm)	ε_{max} ($M^{-1}cm^{-1}$)	Solvent	$\Phi_{fluo.}$	Φ_{Δ}
**5f**	675	700	-	DCM	-	-
	665	698	16000	MeCN	-	0.35
	682	-	-	$CHCl_3$	0.13	-
	679	714	-	DMSO/PBS (4:1)	-	-
	633,703	n.d.	-	DMSO/PBS (1:9)	-	-
**AP5**	696	-	-	DCM	-	-
	677	-	13780	MeCN	-	0.08
	699	-	-	$CHCl_3$	0.001	-
	679	714	-	DMSO/PBS (4:1)	-	-
	639,692	n.d.	-	DMSO/PBS (1:9)	-	-
**6a**	675	706	-	DCM	-	-
	663	700	42000	MeCN	-	0.47
	676	-	-	$CHCl_3$	0.23	-
	679	718	-	DMSO/PBS (4:1)	-	-
**AP6**	693	-	-	DCM	-	-
	677	-	37520	MeCN	-	0.05
	696	-	-	$CHCl_3$	0.001	-

	683	718	-	DMSO/PBS (4:1)	-	-

n.d.: not detected.

2.1 Fluorescence Quantum Yield and Singlet Oxygen Quantum Yield Determination

In the calculation of fluorescence quantum yield, zinc phthalocyanine was employed as reference (excited at 650 nm in pyridine, Φ_{fluo} =0.3)[2]. BODIPY based compounds were dissolved in chloroform. Then, they were excited at 650 nm and their fluorescence values recorded. The fluorescence quantum yield was calculated using the equation shown below.

$$\Phi_x = \Phi_{st} \text{ x } (A_{st}/A_x) \text{ x } (F_x/F_{st}) \text{ x } (\eta_x^2/\eta_{st}^2)$$

In the equation for fluorescence quantum yield, Φ_x is the fluorescence quantum yield of sample, Φ_{st} denotes the fluorescence quantum yield of reference, A is absorbance value at the excitation wavelength, F is the area of fluorescence spectrum with spectral correction for the reference and unknown sample, η represents refractive index for reference and sample.

Indirect method was employed to determine the singlet oxygen quantum yields of the synthesized compounds and estimated values were given in Table 1. DPBF was utilized as the singlet oxygen scavenger and indicator. The original absorbance around 410 nm of the mixture of the compounds with DPBF in acetonitrile was adjusted around 0.8-1 and the $^1O2$ quantum yields were measured by monitoring the accumulative changes of the MeCN solution in the absorbance at 410 nm as the photo-oxidation of the DPBF continued. The absorbance was recorded after every irradiation period (λ_{irr} = 660 nm). Methylene blue was used as the reference (Φ_Δ = 0.53 in ethanol)[3]. All the photosensitizers were employed in MeCN. The quantum yields were calculated according to the following equations,

$$\boldsymbol{\Phi}_{\boldsymbol{\Delta},s} = \boldsymbol{\Phi}_{\boldsymbol{\Delta},r} \text{ x } (S_s \text{ x } F_r)/ (S_r \text{ x } F_s) \text{ where } F= 1-10^{-OD \text{ (at 630nm)}}$$

where *S* denotes the calibrated slope of the linear fit of the cumulative changes of the absorbance at 410 nm *vs.* the cumulative irradiation time. *F* stands for the absorption correction factor. The superscript "*s*" refers to the samples, and "*r*" refers to the reference.

2.2 Additional Absorbance and Fluorescence Spectra

The stock solutions for molecules **5f**, **AP5**, **6a** and **AP6** were prepared in acetonitrile. In the absorbance and fluorescence measurements, the concentration for molecules was adjusted to $1x10^{-5}$ M. Acetonitrile and dichloromethane were used as solvents in the measurements of **AP5**, **5f**, **AP6**, and **6a**.

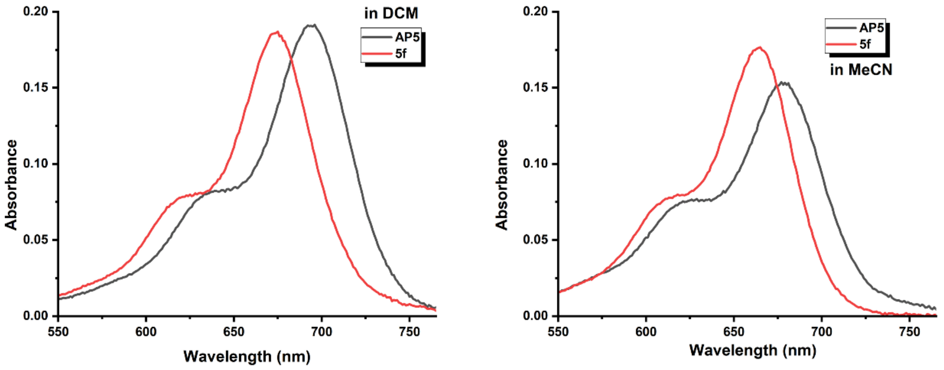

Figure S1. Absorbance spectra of **AP5** (10^{-5} M) and **5f** (10^{-5} M) in different solvents (dichloromethane, acetonitrile).

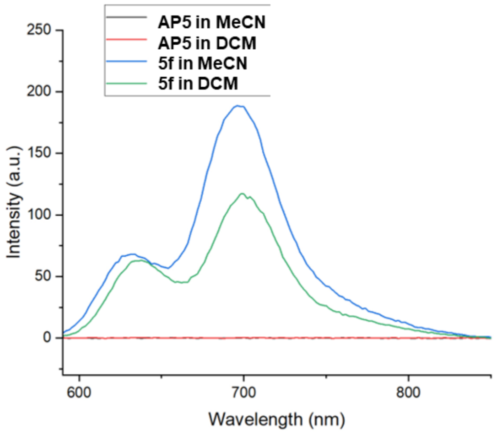

Figure S2. Fluorescence spectrum of **AP5** (10^{-5} M) and **5f** (10^{-5} M) in different solvents (dichloromethane, acetonitrile) (exc. at 580 nm).

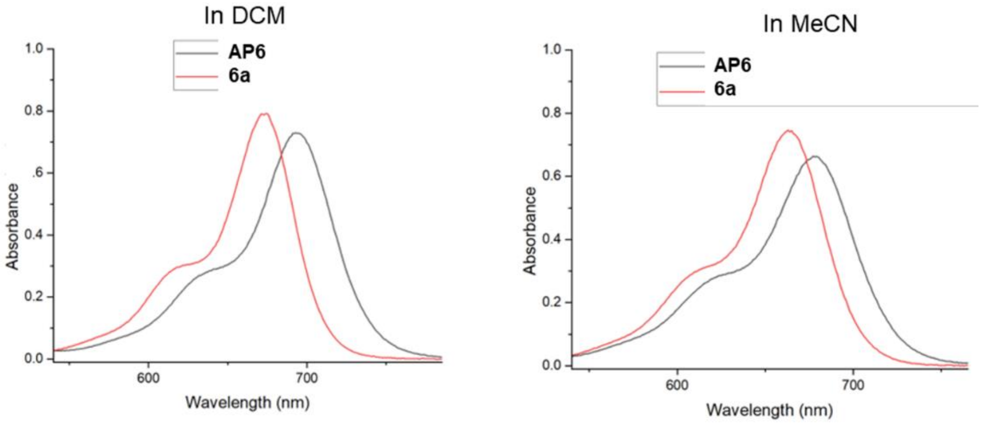

Figure S3. Absorbance spectra of **AP6** (10^{-5} M) and **6a** (10^{-5} M) in different solvents (dichloromethane, acetonitrile).

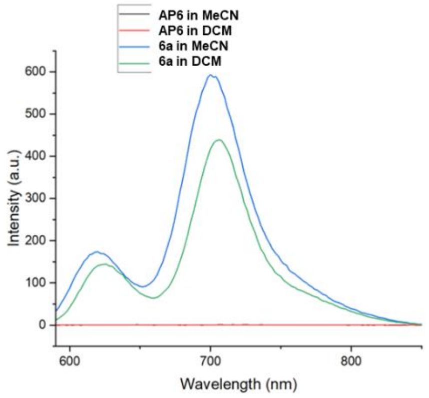

Figure S4. Fluorescence spectrum of **AP6** (10^{-5} M) and **6a** (10^{-5} M) in different solvents (dichloromethane, acetonitrile) (exc. at 580 nm).

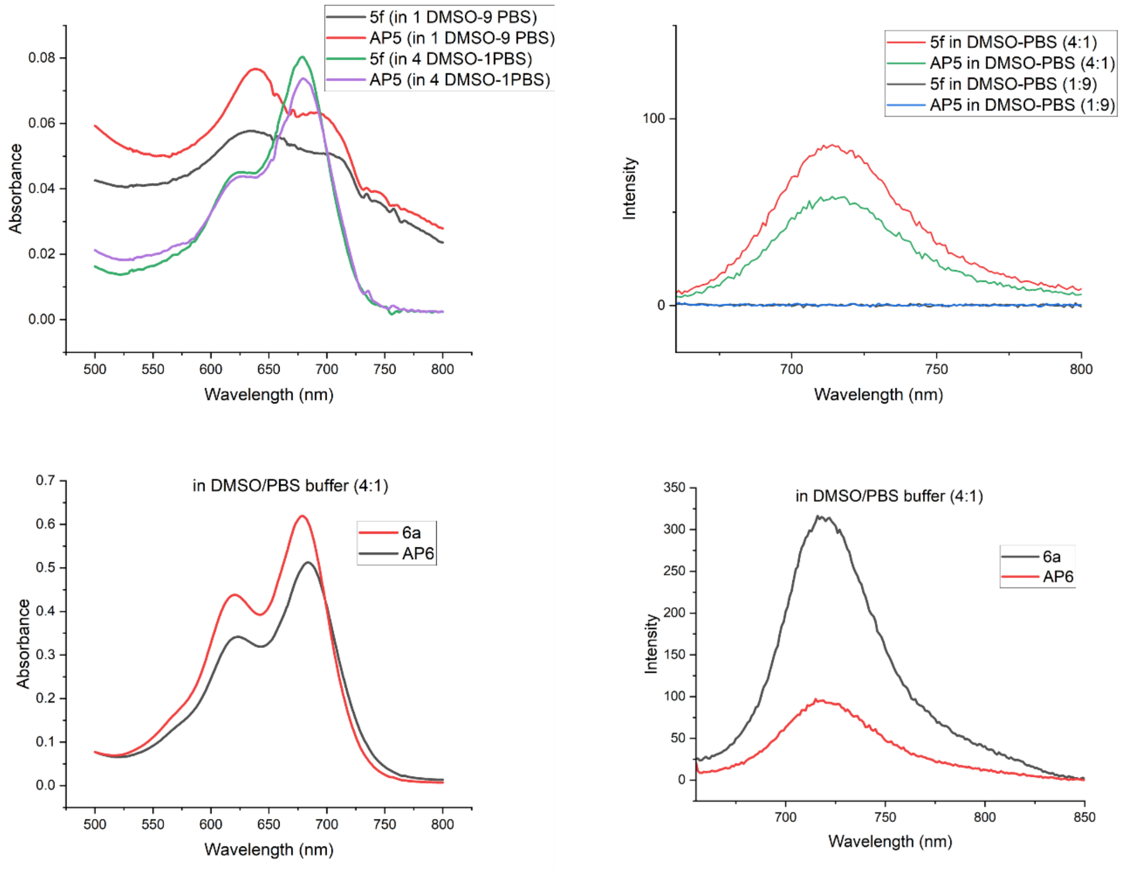

Figure S5. Absorbance and fluorescence spectra of final compounds in DMSO/PBS buffer (4:1 and 1:9).

2.3 Metal Titration Experiments:

To determine the ability of metals to form complexes with molecules **5f** and **6a**, titration experiments were carried out using $Zn(ClO_4)_2.6H_2O$ and $Cu(NO_3)_2.3H_2O$. The stock solutions of **5f** and **6a** were prepared in acetonitrile. The stock solutions for $Zn(ClO_4)_2.6H_2O$ and

$Cu(NO_3)_2.3H_2O$ were also prepared in acetonitrile. In order to determine the most effective complex formation, Cu(II) and Zn(II) were added gradually to the 10^{-5} M solutions of **5f** and **6a**. During the additions, fluorescence and absorption of molecules were recorded.

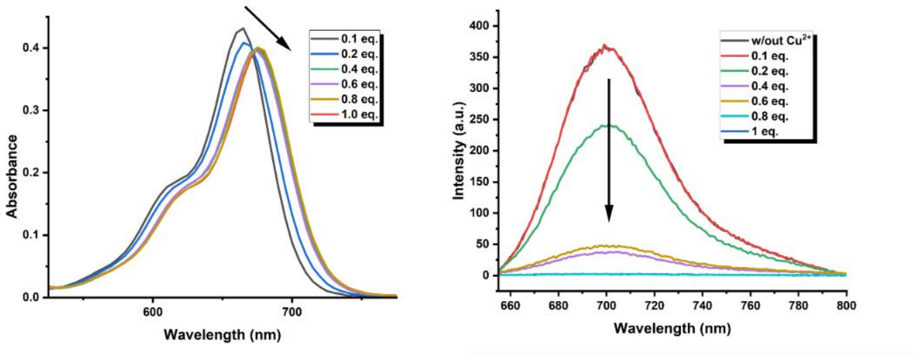

Figure S6. Absorbance (left) and fluorescence (right) spectra of **5f** during metal titration experiment with Cu(II).

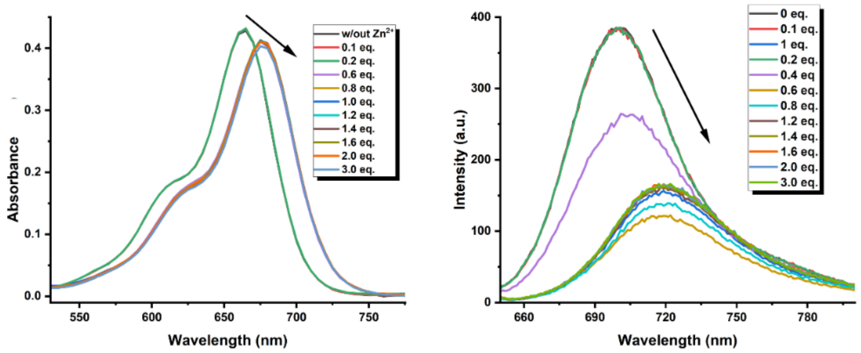

Figure S7. Absorbance (left) and fluorescence (right) spectra of **5f** during metal titration experiment with Zn(II).

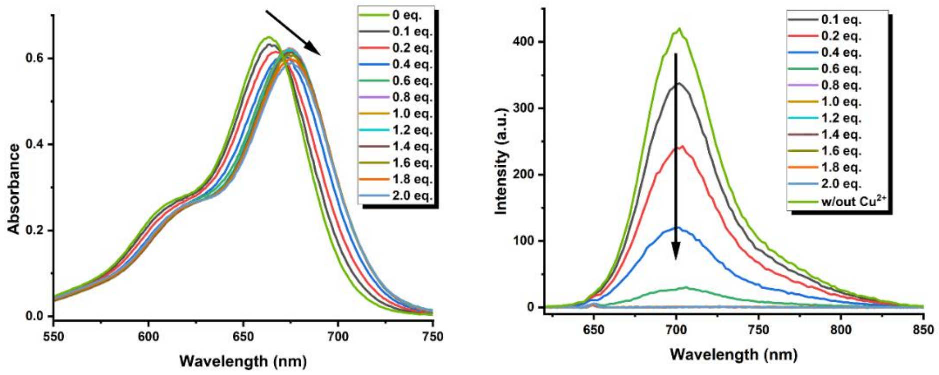

Figure S8. Absorbance (left) and fluorescence (right) spectra of **6a** during metal titration experiment with Cu(II).

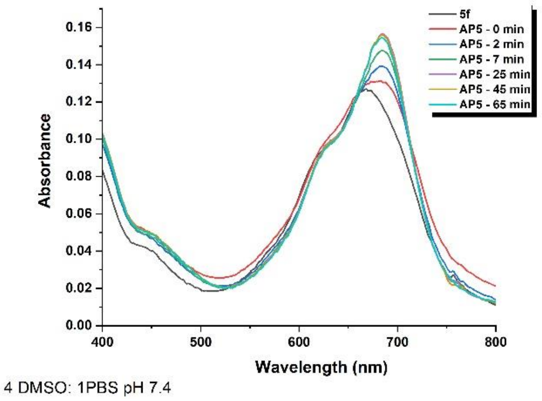

Figure S9. AP5 complex stability test with time; addition of 1 eq. $Cu(NO_3)_2$ to **5f** in aqueous media (4 DMSO: 1 PBS, pH 7.4)

2.4 Nitric oxide addition experiments:

To detect the effect of nitric oxide on the Cu^{2+} complexed molecules **AP5** and **AP6**, experiments were performed using Diethylamine NONOate (DEA NONOate, Cayman Chemicals). These experiments were carried out according to the procedure given in the reference.[4] The stock solution of DEA NONOate was prepared in 0.01 M NaOH (or in PBS in Figure 3b) and then $5x10^{-5}$ M solutions of **AP5** and **AP6** were also prepared in acetonitrile. After adding 8 eq. DEA NONOate to the $5x10^{-5}$ M solutions of **AP5** and **AP6**, fluorescence spectra of mixtures were recorded to determine the enhancement in the fluorescence.

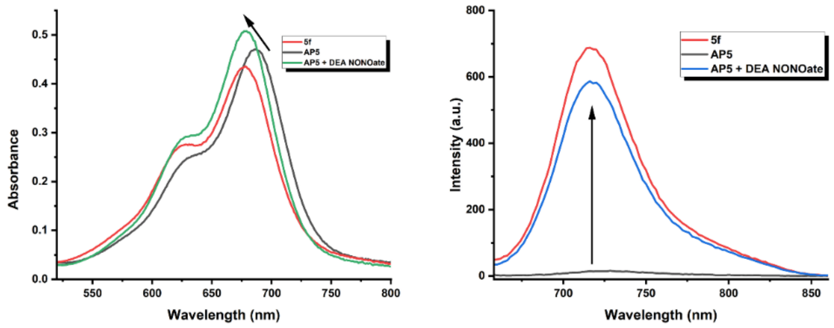

Figure S10. Absorbance and fluorescence spectra of **AP5** during DEA NONOate addition experiment (in MeCN).

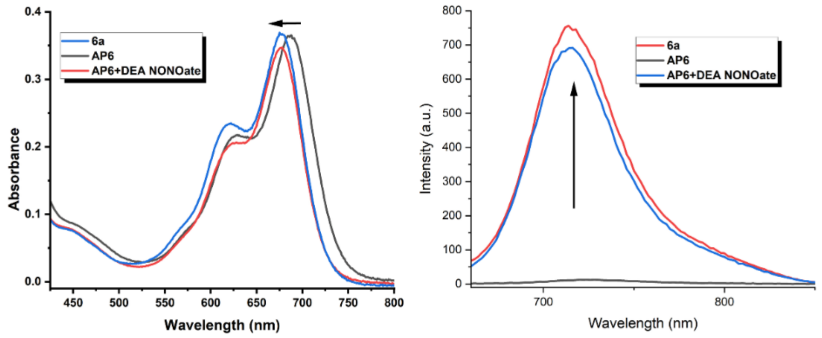

Figure S11. Absorbance and fluorescence spectra of **AP6** during DEA NONOate addition experiment (in MeCN).

2.5 Reactive Oxygen Species (ROS) Determination Experiments:

Trap molecule 1,3-Diphenylisobenzofuran (DPBF) that has an absorption maximum around 410 nm was used as an indicator for the presence of ROS. When the ROS present in the medium, absorbance of DPBF at 411 nm decreases and the singlet oxygen production could be monitored. For prodrugs or photosensitizers both organic and aqueous solvent systems were employed. For molecule **AP5** acetonitrile, DMSO-PBS (4:1), DMSO-PBS (1:9) solvents were used. The stock solutions of **AP5**, **AP6** were prepared in acetonitrile. For **AP6**, acetonitrile and DMSO-PBS (4:1) solvents were employed. The LED used in the experiments has emission maximum wavelength of 660 nm. Experiments were performed at the same concentration of molecules. After keeping the solutions under dark for a few minutes, molecules were illuminated for 1-minute intervals.

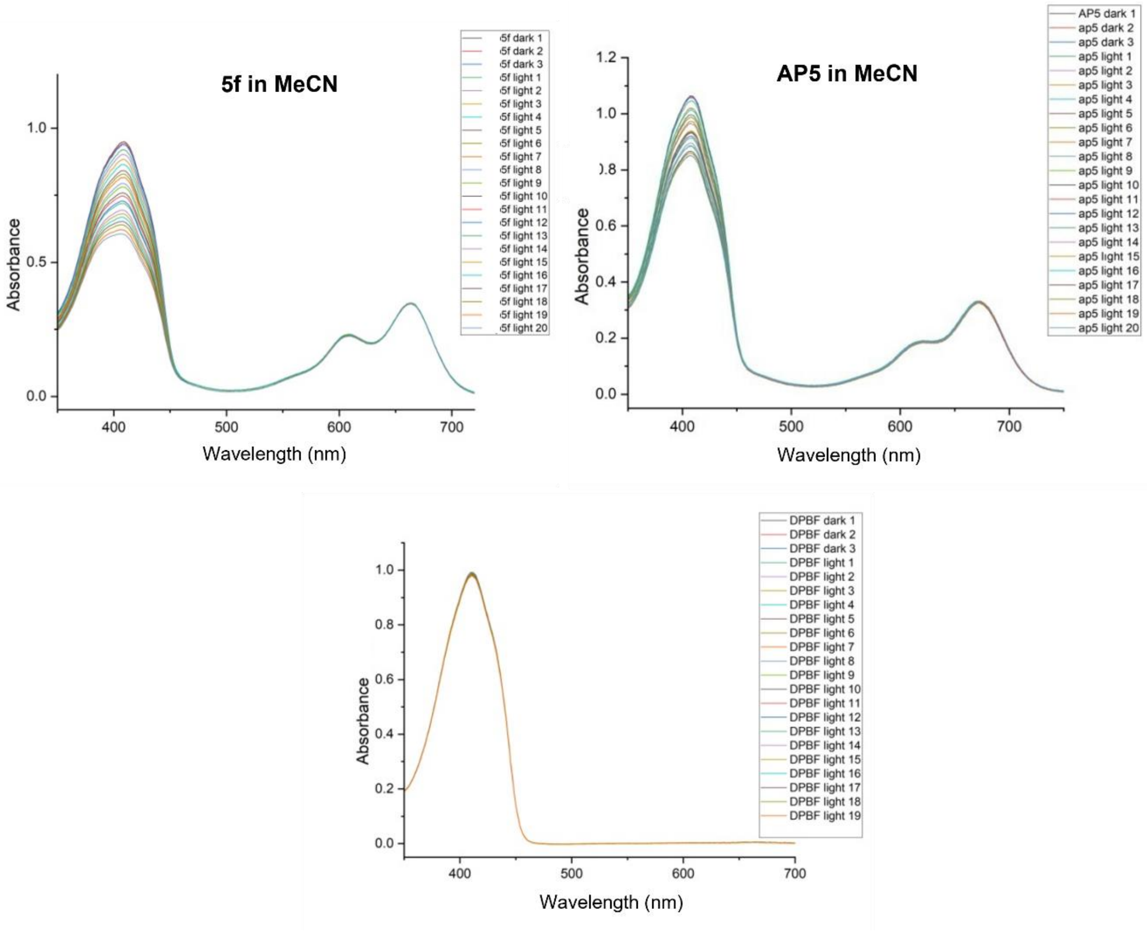

Figure S12. Changes in Absorbance spectra of ROS trap DPBF in the presence of **5f (upper left), AP5 (upper right)** and control (bottom, without PS, DPBF only) in acetonitrile under dark and irradiation with LED.

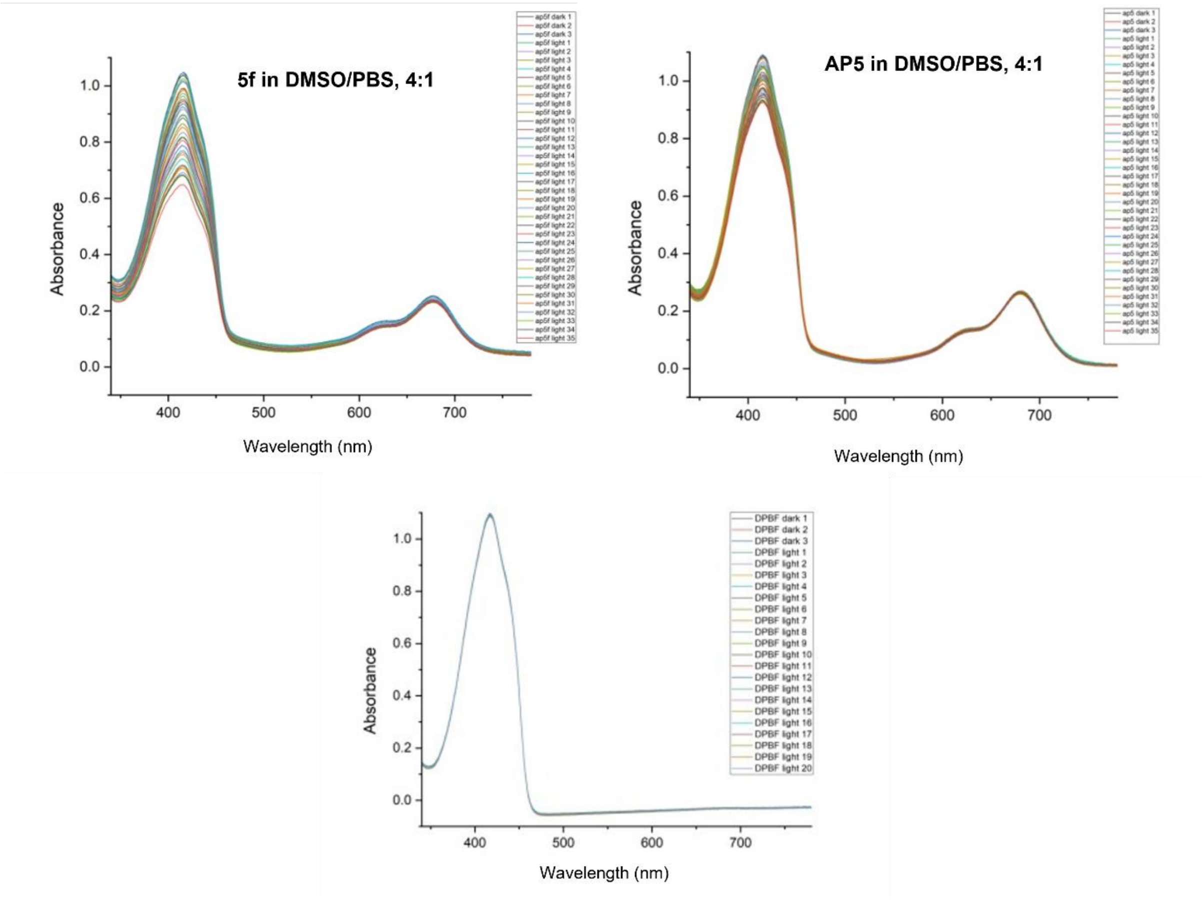

Figure S13. Changes in Absorbance spectra of ROS trap DPBF in the presence of **5f** (upper left), **AP5** (upper right) and control (bottom, without PS, DPBF only) in **DMSO-PBS (4:1, 10^{-5}M)** solvent under dark and irradiation with LED.

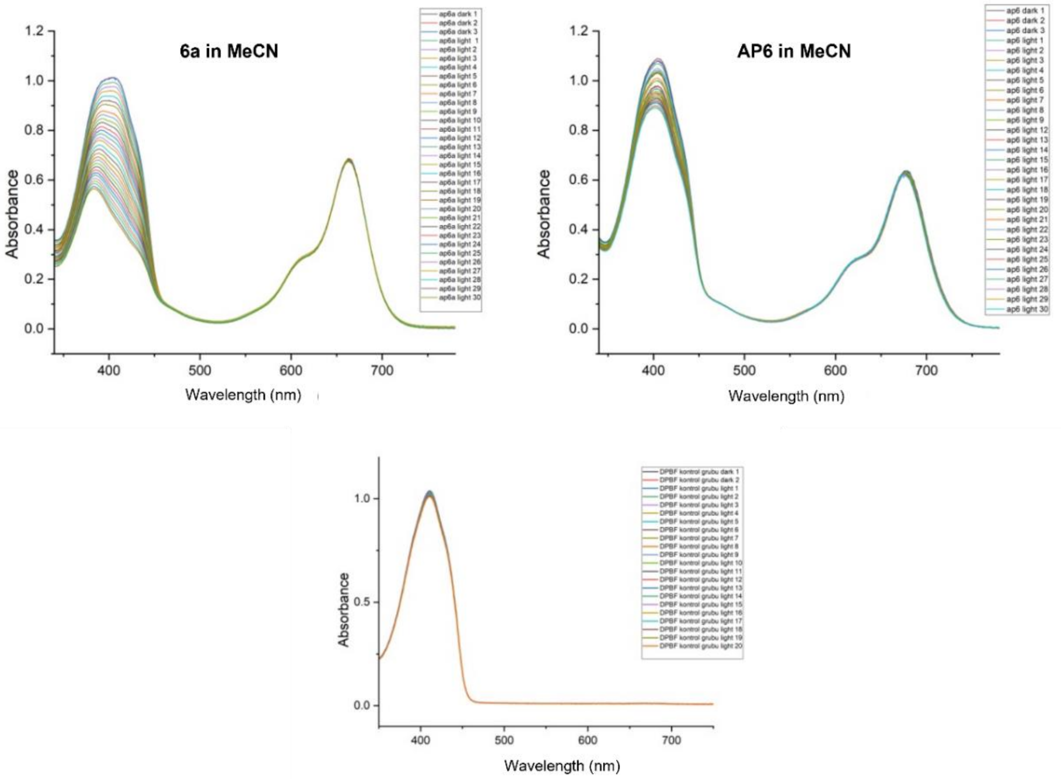

Figure S14. Changes in Absorbance spectra of ROS trap DPBF in the presence of **6a** (upper left)**, AP6** (upper right) and control (bottom, without PS, DPBF only) in **acetonitrile** solvent under dark and irradiation with LED.

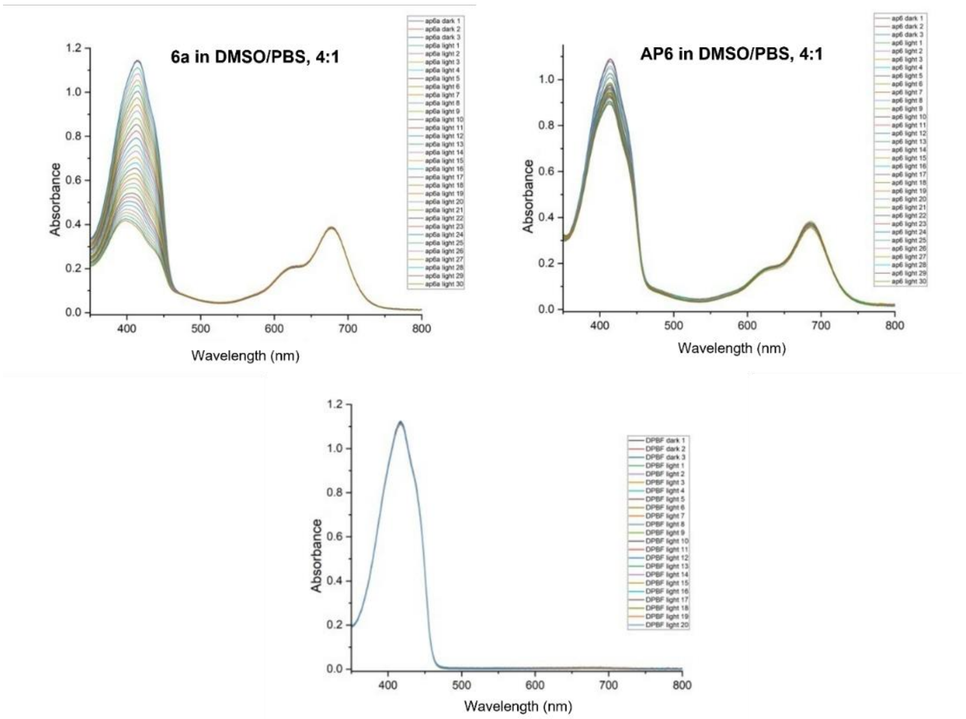

Figure S15. Changes in Absorbance spectra of ROS trap DPBF in the presence of **6a** (upper left), **AP6** (upper right) and control (bottom, without PS, DPBF only) in **DMSO-water (4:1)** under dark and irradiation with LED.

2.6 ROS Determination Experiments upon NO activation:

Similar conditions were employed for NO addition as explained above.

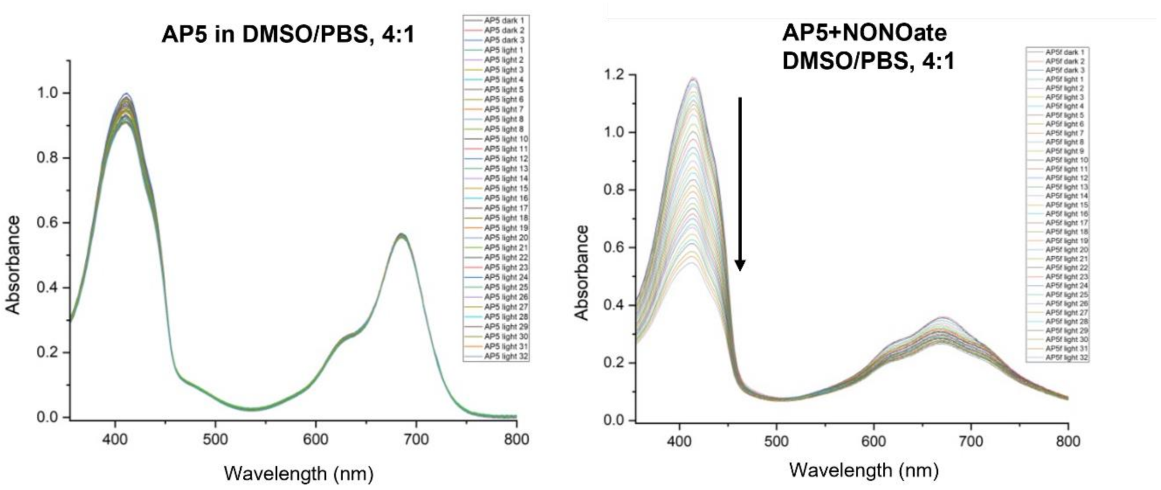

Figure S16. Changes in absorbance spectra of ROS trap DPBF in the presence of **AP5** (left) and **AP5+DEA NONOate** (right) under dark and irradiation with LED **[DMSO-PBS (4:1)].**

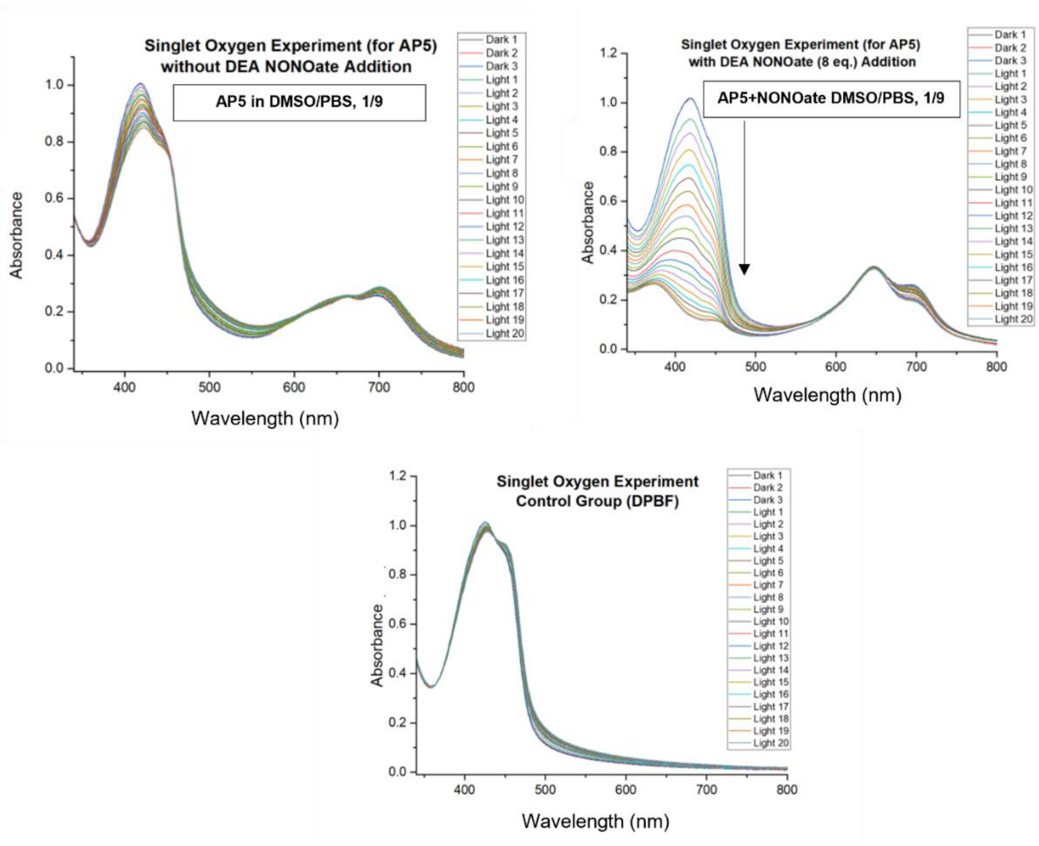

Figure S17. Changes in Absorbance spectra of **ROS** trap DPBF in the presence of AP5 (upper-left), AP5+DEA NONOate (upper-right), control (bottom, without PS, bottom) under dark and irradiation with LED [DMSO-PBS (1:9)]. 8 eq. of DEA NONOate was added.

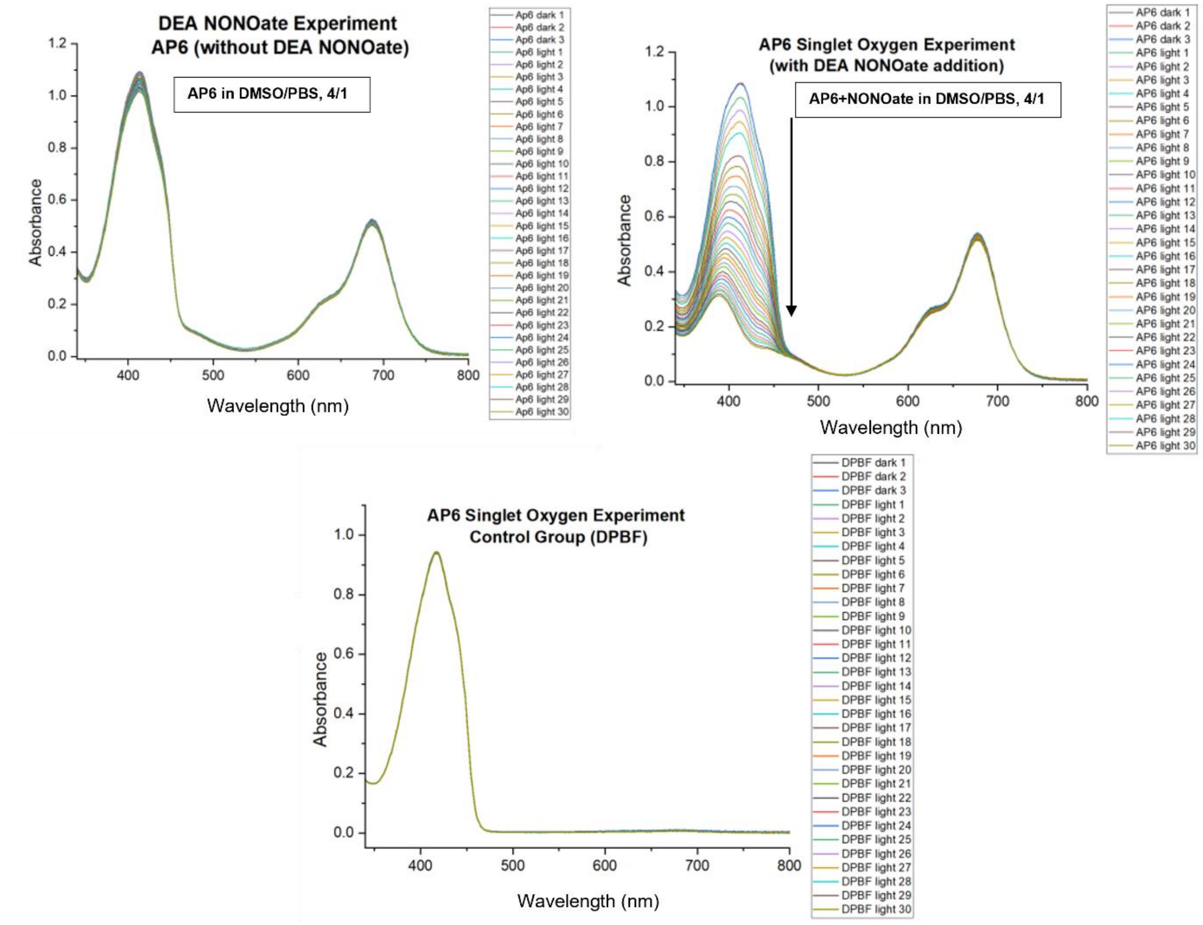

Figure S18. Changes in Absorbance spectra of ROS trap DPBF in the presence of **AP6** (up-left), **AP6+DEA NONOate** (up-right), control (without PS, bottom) under dark and irradiation with LED **[DMSO-PBS (4:1)]**. 8 eq. of DEA NONOate was added.

2.7 Interaction of AP5 and AP6 with Cysteine and Glutathione:

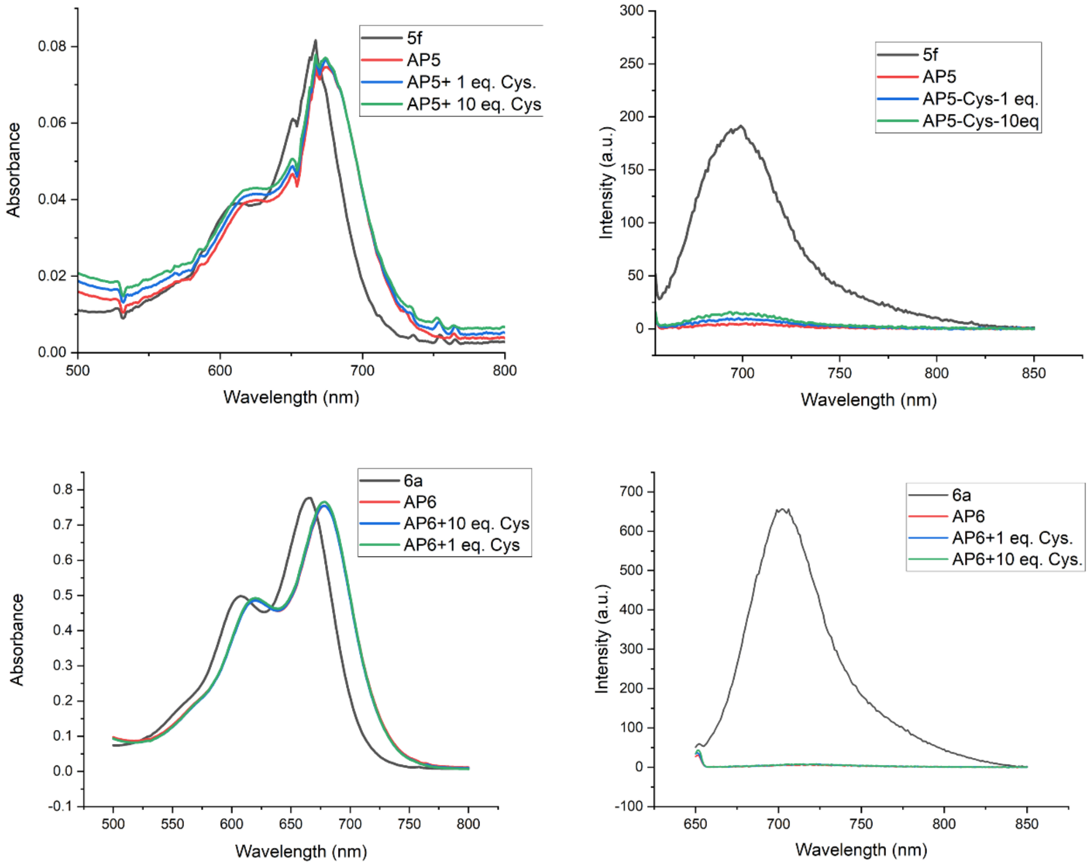

Figure S19. Interaction of Cysteine with **5f**, **AP5**, **6a** and **AP6**, determined by absorbance and fluorescence spectrometry in MeCN.

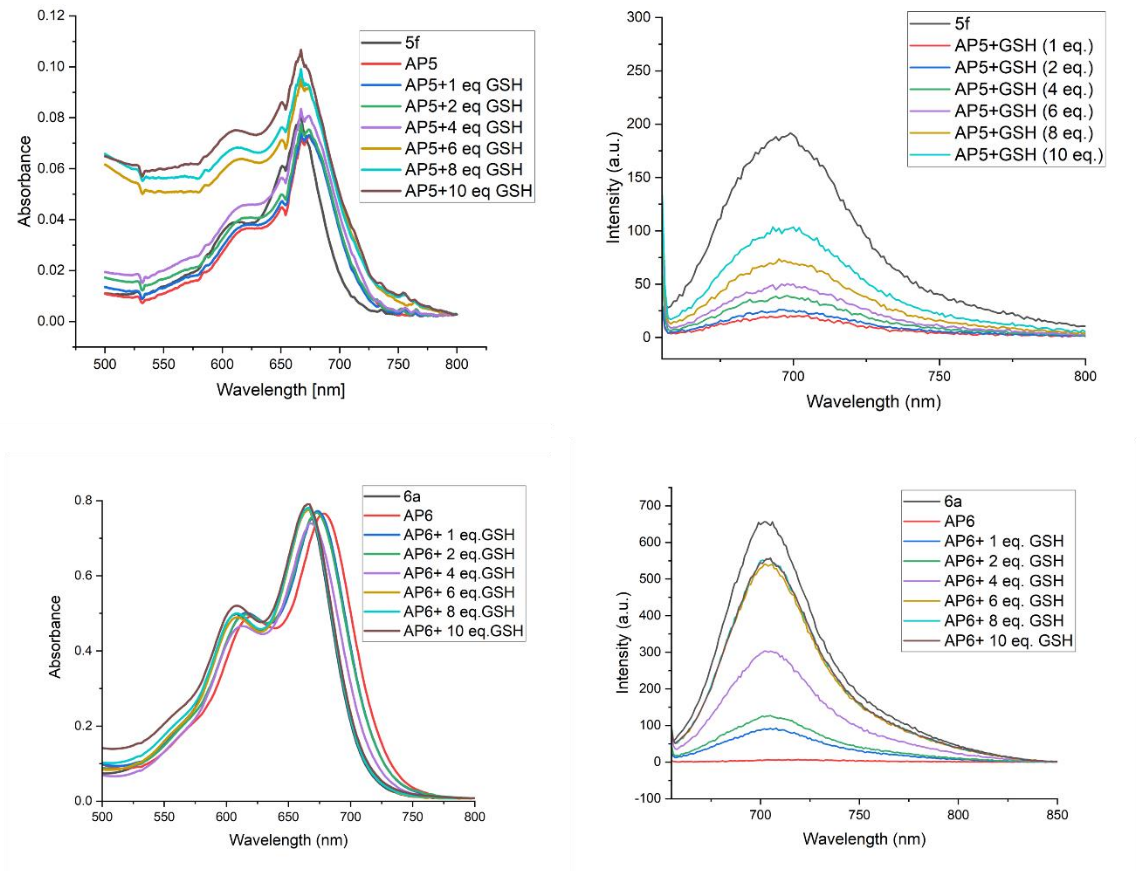

Figure S20. Interaction of Glutathione with **5f**, **AP5**, **6a** and **AP6**, determined by absorbance and fluorescence spectrometry in MeCN.

2.8 Interaction of AP5 and AP6 with Quercetin

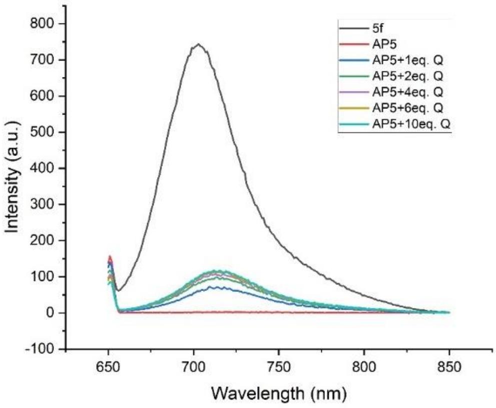

Figure S21. Fluorescence spectra of 5f,AP5 and successive additions of quercetin (Q) to AP5 in MeCN.

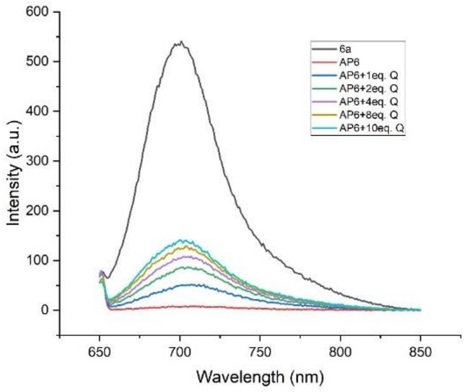

Figure S22. Fluorescence spectra of 6a, AP6 and successive additions of quercetin (Q) to AP6 in MeCN.

3 Cell Culture Experiments and Fluorescence Microscopy Imaging

To test the cytotoxicity of **AP5** and **AP6**, *in vitro* cell culture experiments were carried out. In the cell culture studies, two types of cell lines were used to test the cytotoxicity and activation of the synthesized prodrugs. RAW264.7 cell lines and HeLa cell lines (by addition of a NO source, diethylamine NONOate where appropriate was added) were used.

For HeLa cell culture: 10000 cell/well HeLa cells were seeded to 96 well plate, after one day of incubation, the given concentrations 1 µM of **AP5, AP6 (inactive)** and **5f**, **6a (active)** and **AP5 (or AP6) +** DEA NONOate or $Cu(NO_3)_2$ (for control experiment, Figure S18) were administered to the cells separately. Cells were incubated with PSs for 24 h and old medium was removed, and each well were washed with PBS twice and 0.5 mM of NONOate was added to **AP5** and **AP6** groups (where appropriate) and incubated for 30 mins. After incubation, wells that were added NONOate were washed with PBS twice and new medium was added. Then, phototoxicity was initiated with 1h red light exposure (660 nm LED array, 0.03 W/cm^2, 108 J/cm^2). Similar replicates of the test groups were kept under dark condition as dark control groups. After irradiation, cells were incubated for 24 hours, and cell viability was quantified with the MTT cytotoxicity test. Same experimental sets were prepared for microscopic visualization, cellular morphologies were imaged using Zeiss Axio Vert A1 microscope equipped with Axiocam 105 color camera and Zeiss Axio Observer Z1 fluorescent microscope.

For RAW 267.4 cell culture: 10000 cell/well RAW267.4 cells were seeded to 96 well plate, after one day of incubation, the given concentrations 1 µM of **AP5, AP6** and **5f, 6a** were administered to the cells. The process used for HeLa cells was replicated for RAW 267.4 cells, except for the addition of NONOate.

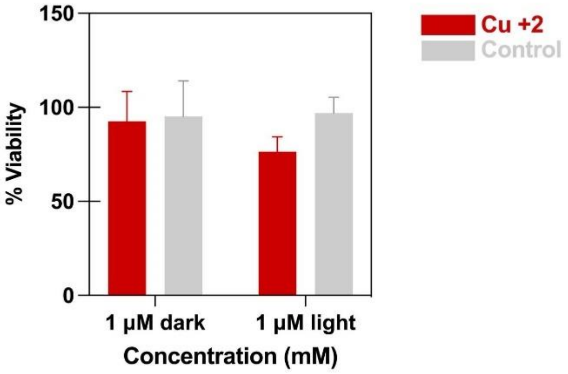

Figure S23. Cellular viability in the presence of Cu^{2+} and with or without radiation.

3.1 Live/Dead Assay:

Elabscience Calcein AM/PI Double Staining Kit was used to visualize live and dead cells. Cells were cultured under the same conditions as described previously. Cells were exposed to red light and incubated for 24 hours post-exposure and staining solution prepared according to the procedure described in the kit was added at 100 µL per well. After incubation at 37 °C for 30 min, imaging was performed by fluorescence microscopy.

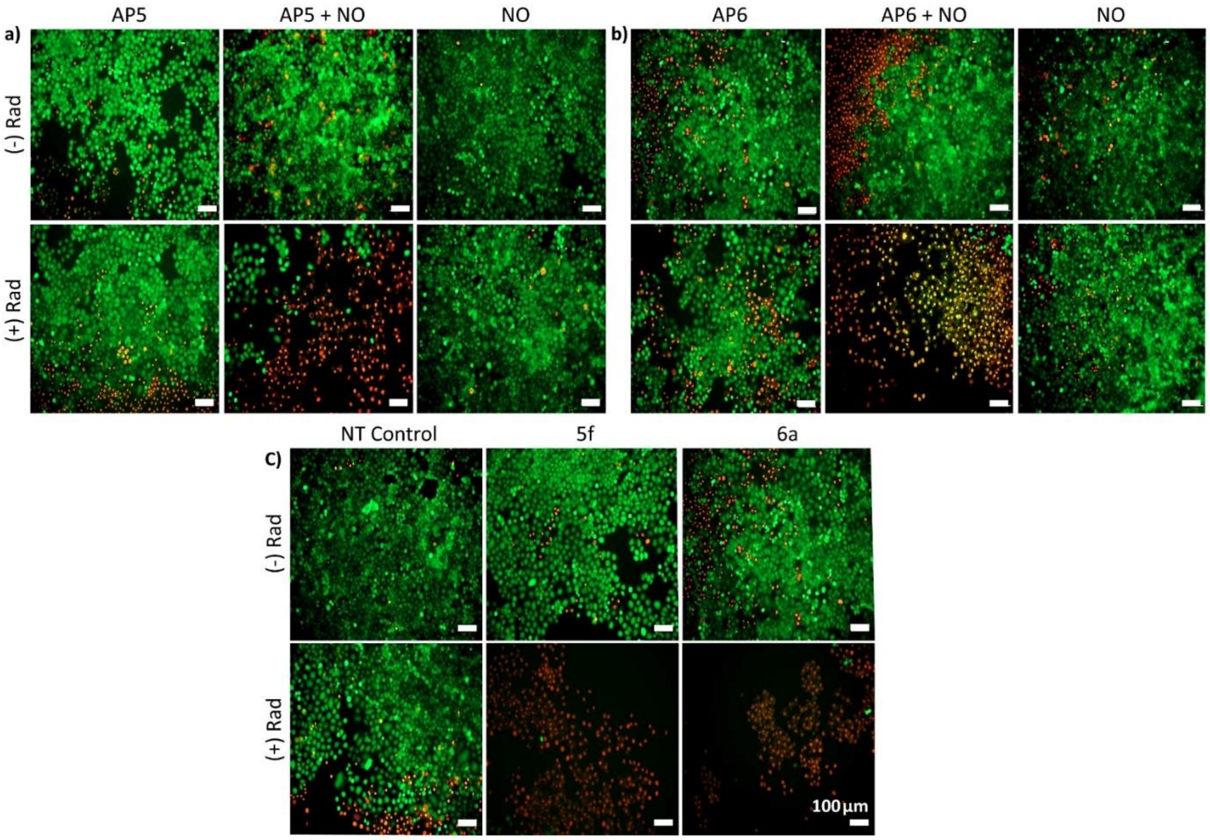

Figure S24. Fluorescence microscopy results of the dark and irradiated groups of the PSs treated with the live/dead assay kit (NT: not treated with PSs).

3.2 ROS Assay :

Elabscience Reactivated Oxygen Species (ROS) Kit based on DCFH-DA was used for ROS assay. For the experiment, 10000 HeLa cells were seeded per well with n = 3 replicates were seeded in two 96-well microplates (illuminated and non-illuminated groups) and incubated for

1 day. After incubation, cells were treated with 1 µM active (5f, 6a), inactive (AP5, AP6), and Cu^{+2}. Cells were incubated with PSs for 6 hours at 37°C. t-BHP (100 µM) solution was added to the positive control groups 1 hour before the end of incubation. Post incubation, the wells were washed twice with PBS. Wells were replaced with 10 µM DCFH-DA solution prepared with colourless DMEM and incubated at 37 °C for 40 min. After incubation, DMEM was discarded, and the remaining DCFH-DA was washed 1 time with PBS buffer. 0.5 mM 20 µL NONOate was added to the groups with NONOate. The light-treated group was incubated under light and the non-light-treated group was incubated in the dark at 37°C for 1 hour. Following light treatment, imaging was performed using a fluorescence microscope.

3.3 Internalization:

Internalization experiments were performed to examine the entry of materials into the cell. For this, 10000 HeLa or RAW264.7 cells were seeded in a 96-well microplate and incubated for one day. After incubation, for HeLa cells 1µM or 100 nM of AP5, AP6, 5f, and 6a were added to the wells separately, and microscope images were taken at 0, 1, 2, 4, and 24 hours. For RAW 264.7 cells 500 nM of agents were employed.

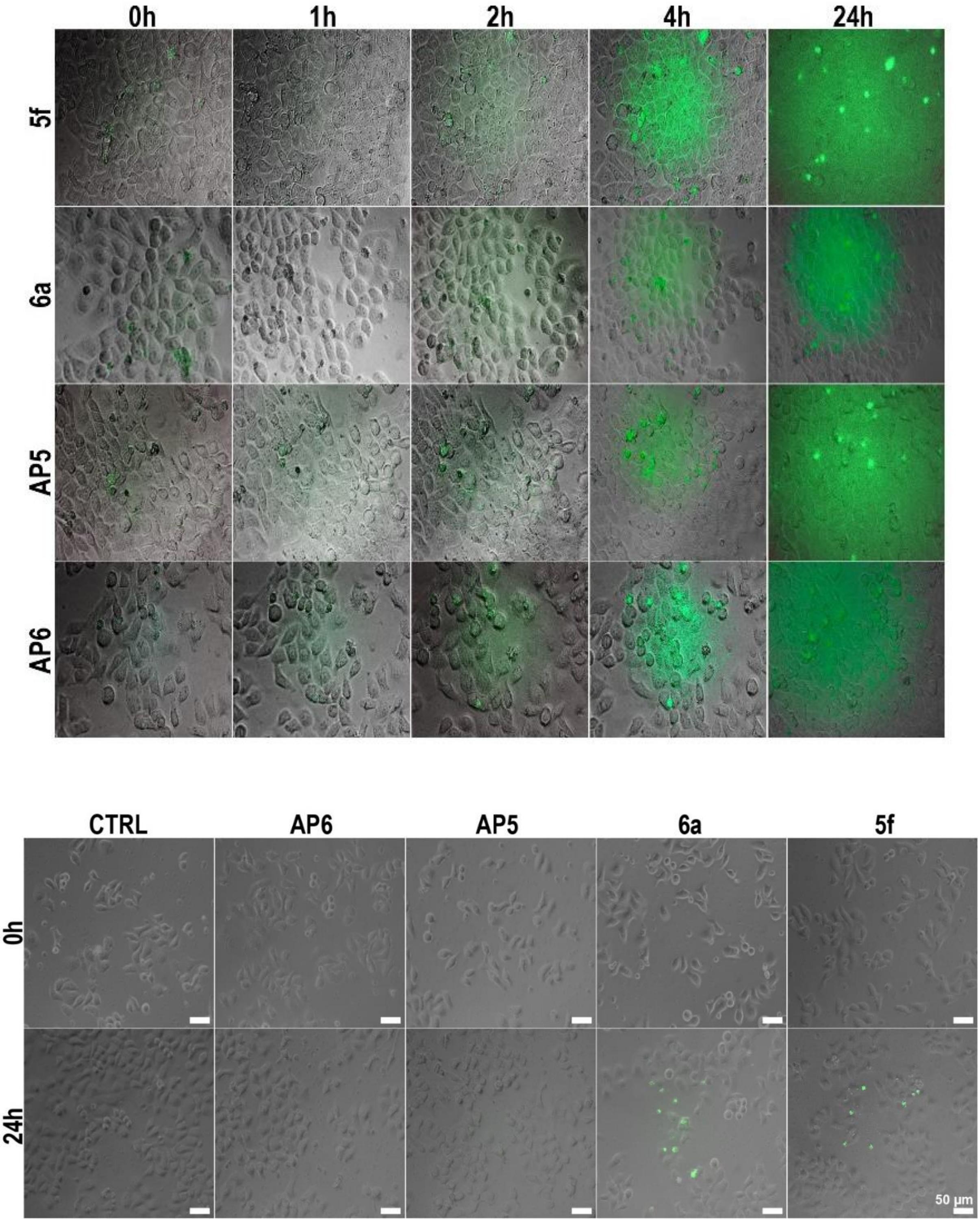

Figure S25. Time-dependent intracellular uptake of substances administered to HeLa cells was demonstrated by merging phase contrast and fluorescence images. 1 µM (upper) and 100 nM (bottom) of PSs were added to the cells.

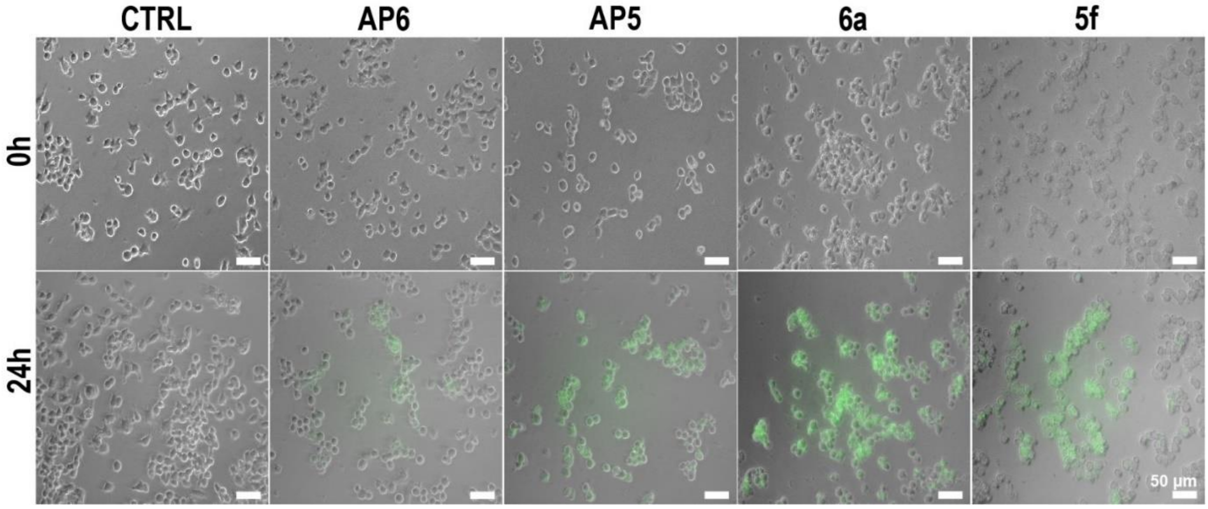

Figure S26. Time-dependent intracellular uptake of substances administered to RAW 264.7 cells was demonstrated by merging phase contrast and fluorescence images. 500 nM of PSs were added to the cells.

3.4 NO Inhibition Control Experiments:

To test the viability of AP5 and 5f, in vitro cell culture experiments were conducted. For the experiment, RAW 264.7 cells were seeded at a density of 20,000 cells/well into 96-well plates with n=3 replicates and incubated for one day. Following the overnight incubation, quercetin was added to the quercetin groups to achieve a final concentration of 25 µM/well, and the cells were incubated with quercetin for 1 hour. Subsequently, all the groups received 500 nanomolar (nM) concentrations of AP5 (inactive) or 5f (active) and were incubated for one day. After one-day incubation, the illuminated groups were exposed to red light for 2 hours and then incubated for an additional 24 hours post-irradiation. Cell viabilities were determined colorimetrically using the 3-(4,5-dimethylthiazol-2-yl)-2,5-diphenyltetrazolium bromide (MTT) cytotoxicity assay.

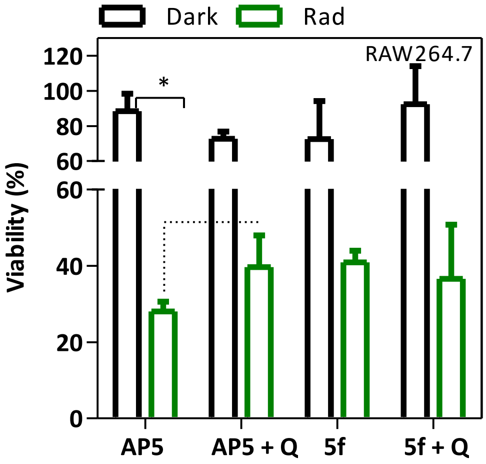

Figure S27. Viability of RAW 264.7 cells in the absence/presence of a NO quencher, quercetin (Q).

4 ^{1}H and ^{13}C NMR Data

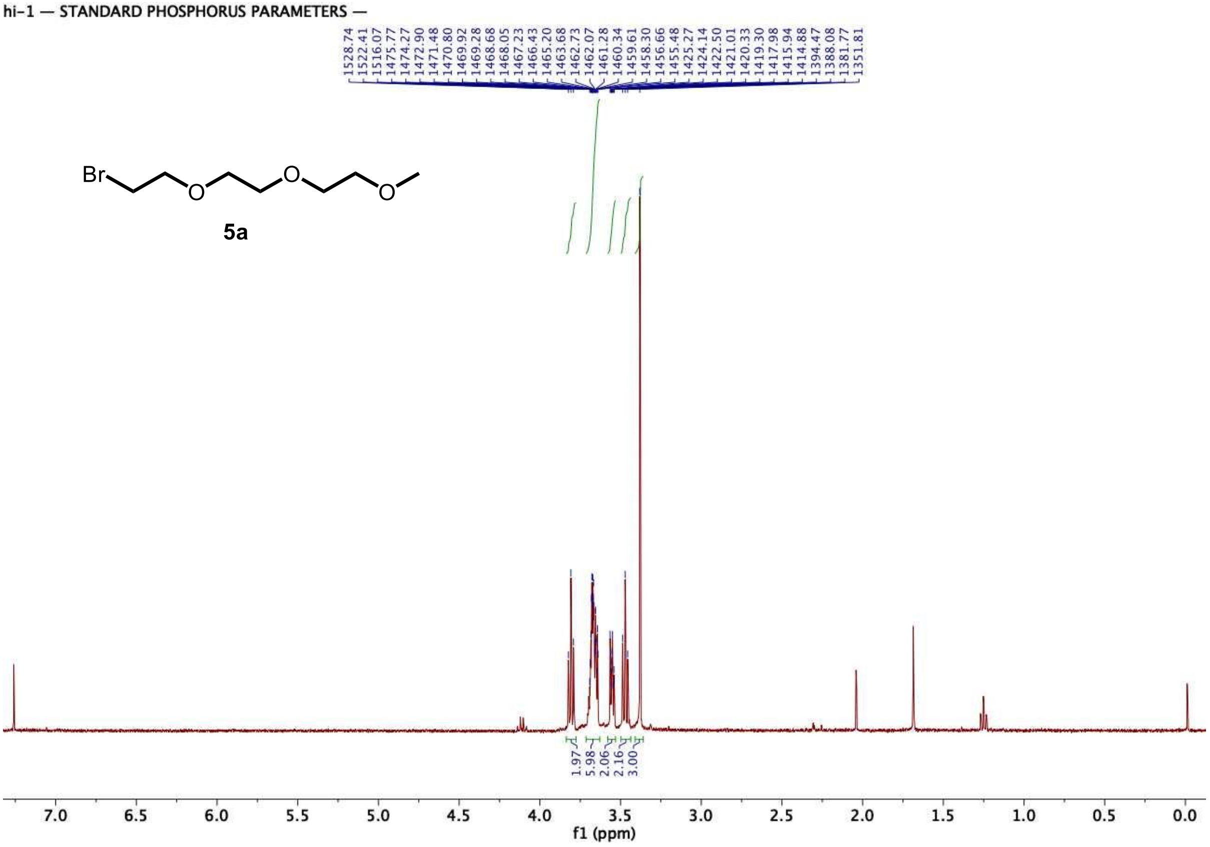

Figure S28. ^{1}H NMR spectra of 5a (400 MHz, $CDCl_3$).

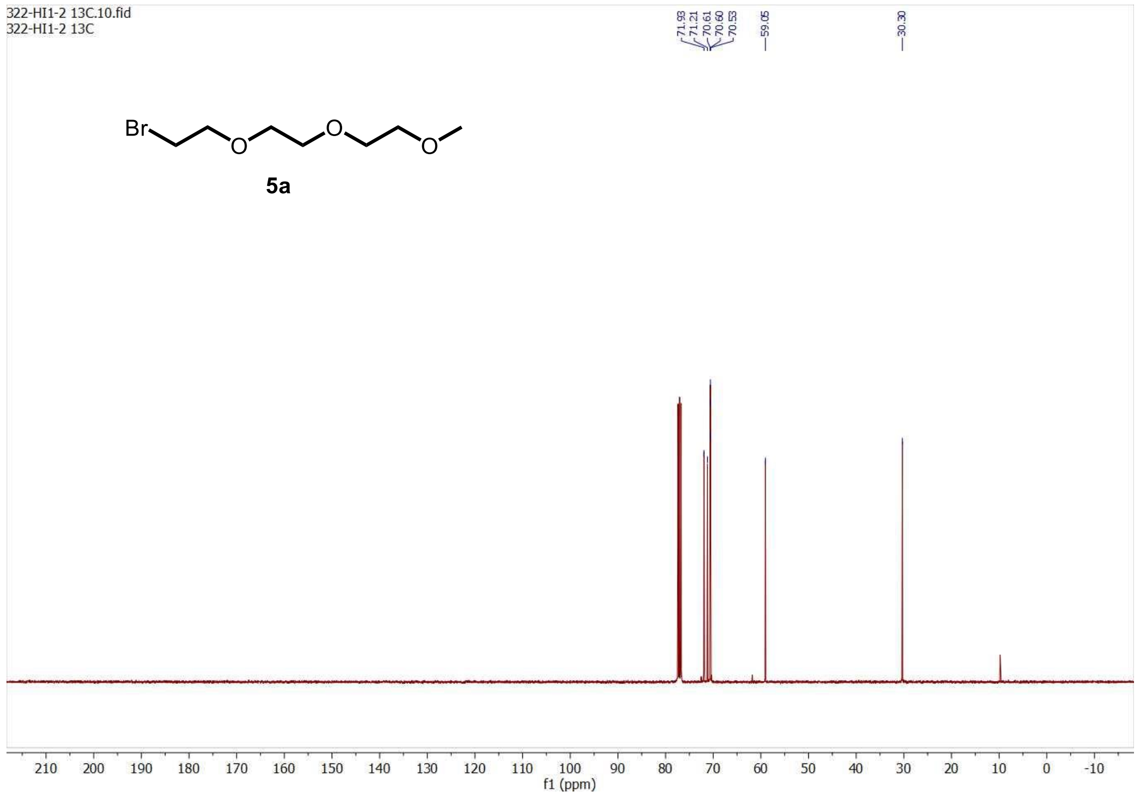

Figure S29. ^{13}C NMR spectra of 5a (100 MHz, $CDCl_3$).

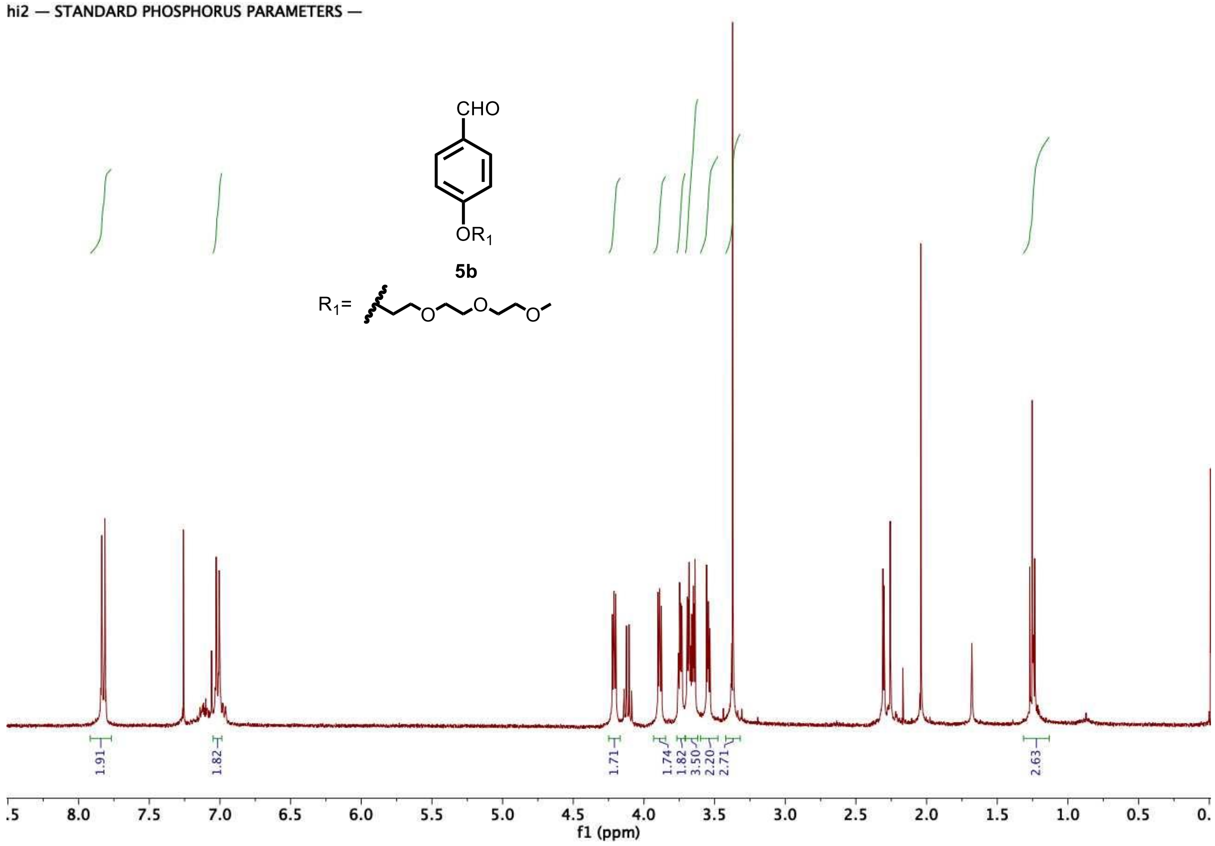

Figure S30. ^{1}H NMR spectra of 5b (400 MHz, $CDCl_3$).

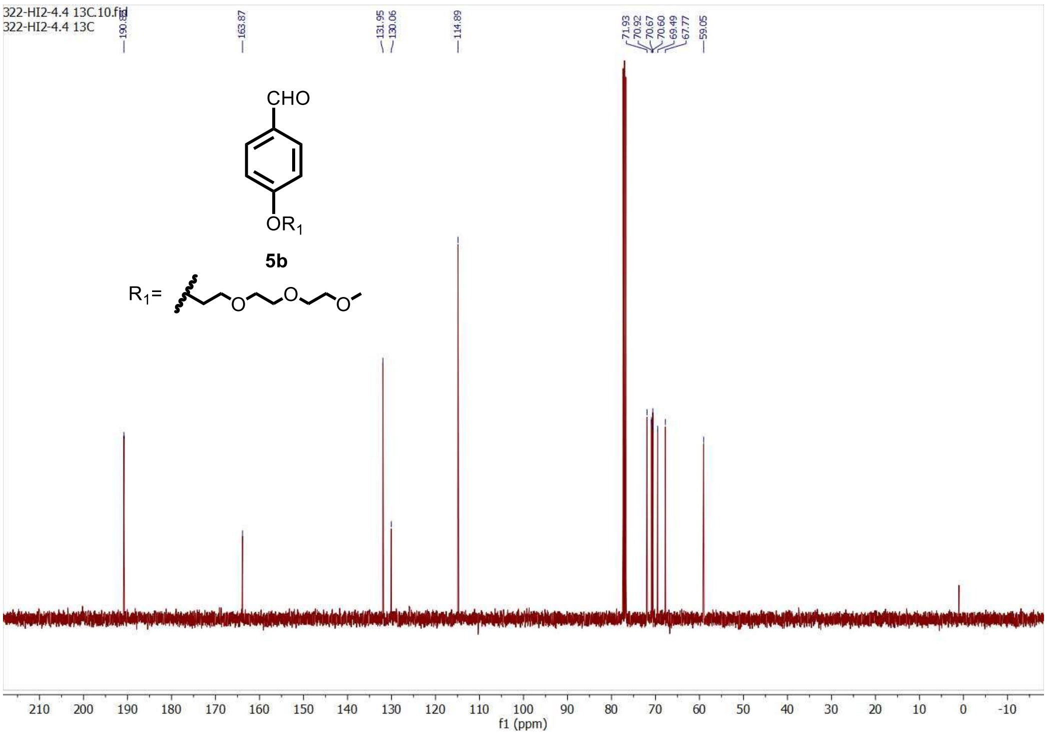

Figure S31. ^{13}C NMR spectra of 5b (100 MHz, $CDCl_3$).

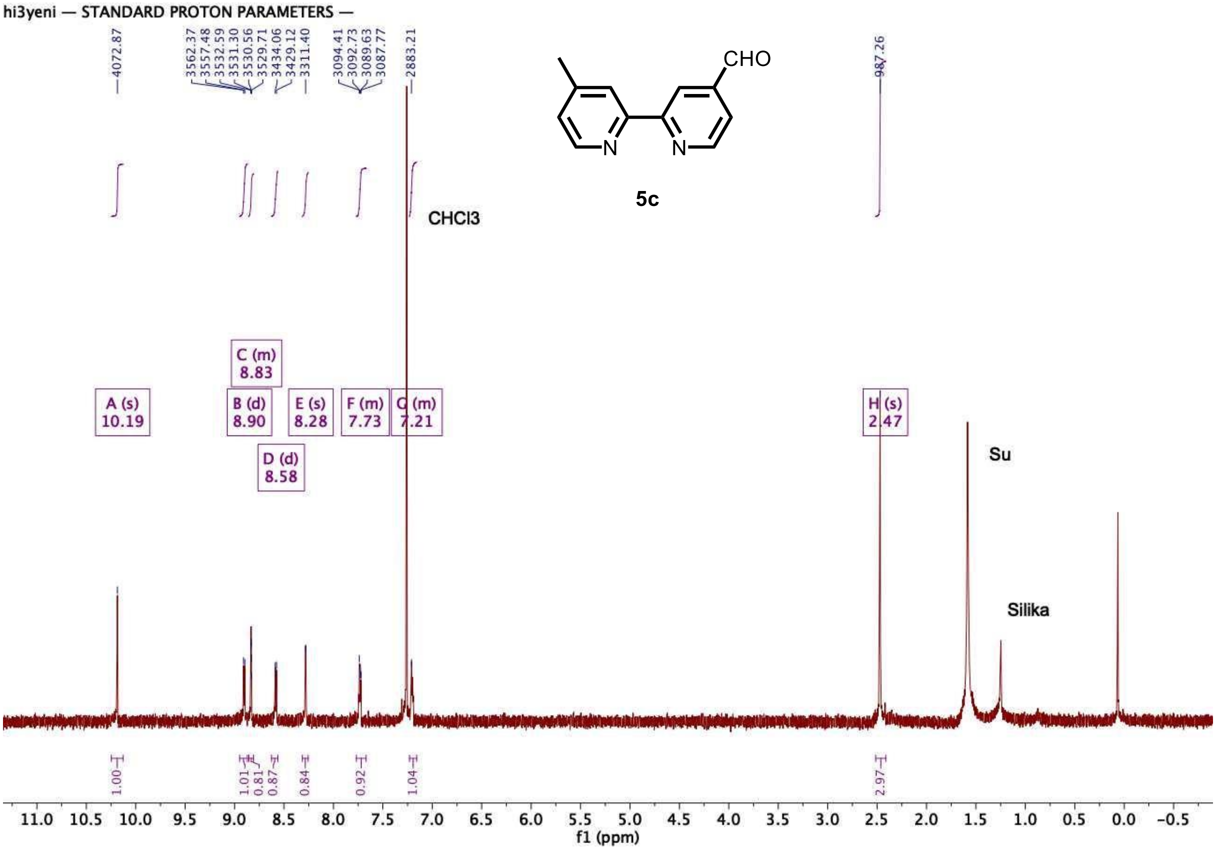

Figure S32. ^{1}H NMR spectra of 5c (400 MHz, $CDCl_3$).

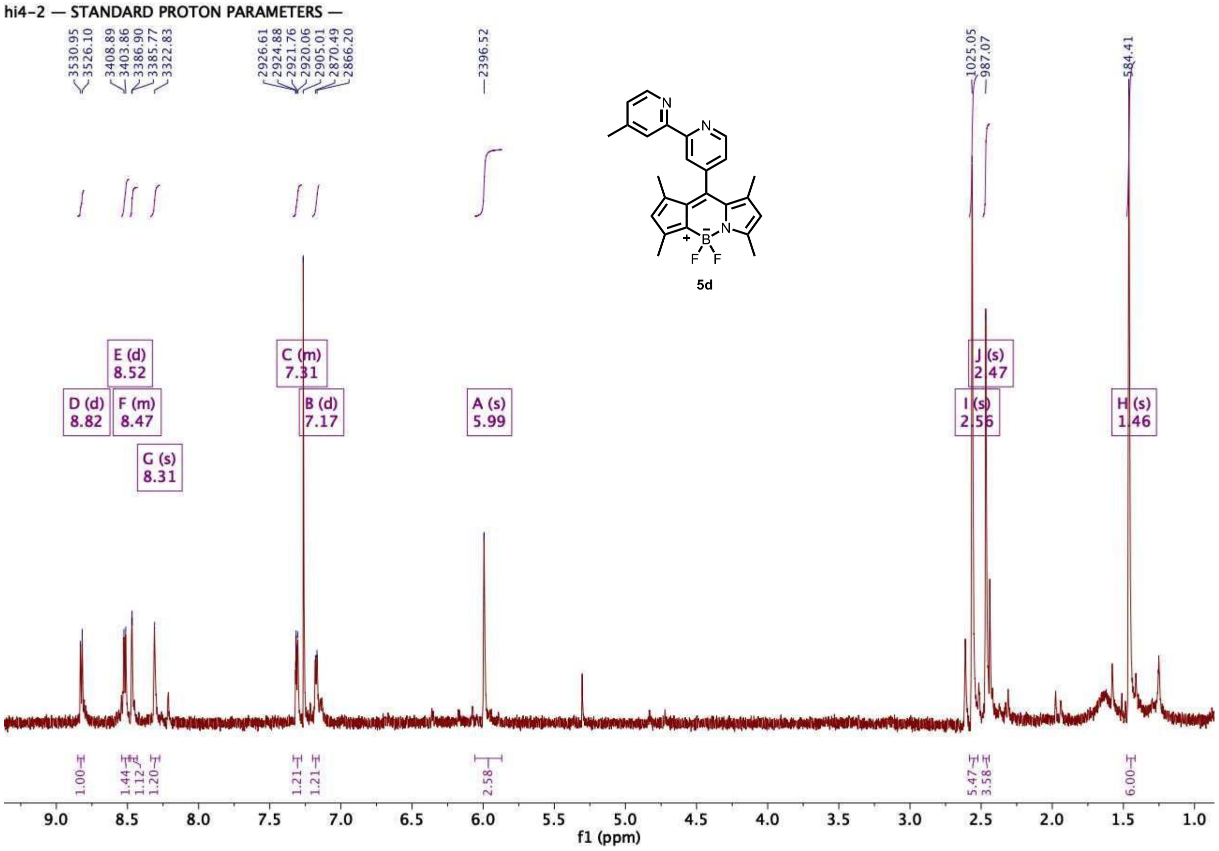

Figure S33. ^{1}H NMR spectra of 5d (100 MHz, $CDCl_3$).

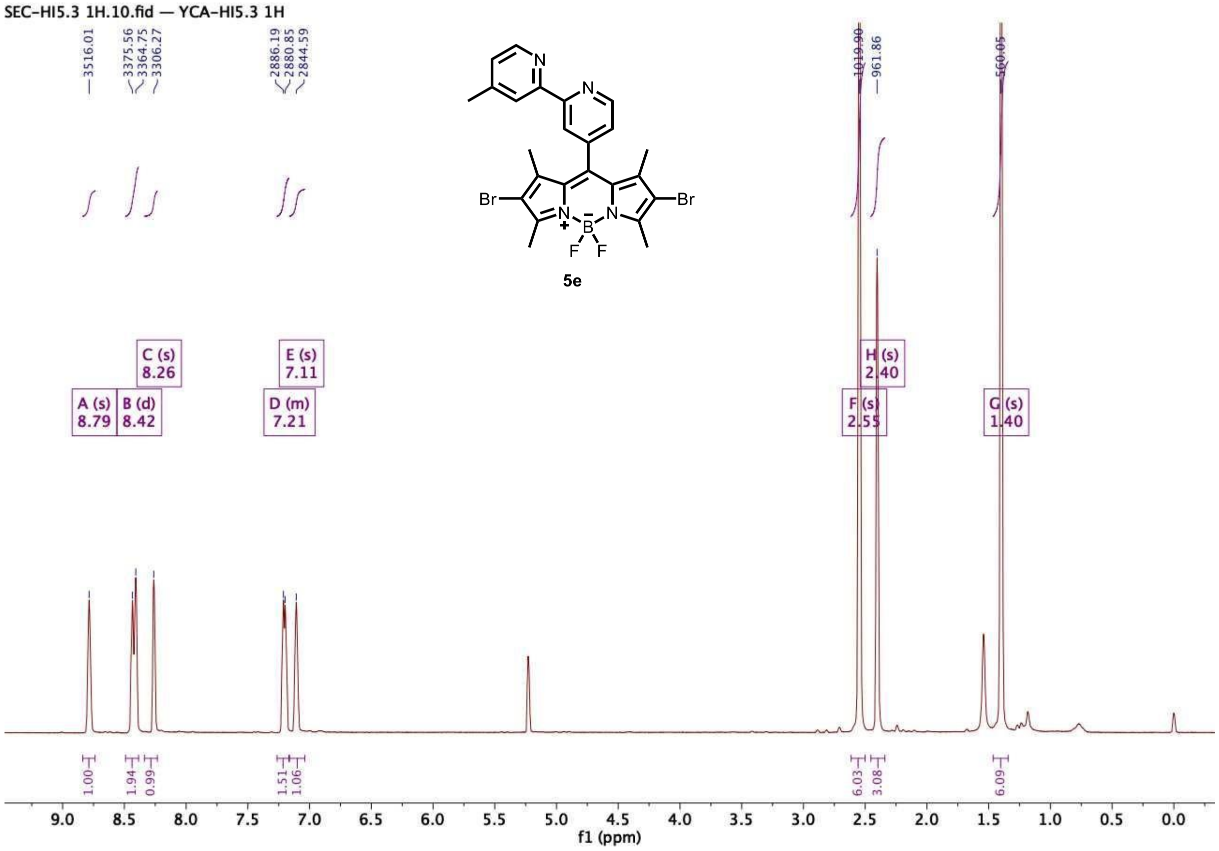

Figure S34. ^{1}H NMR spectra of 5e (400 MHz, $CDCl_3$).

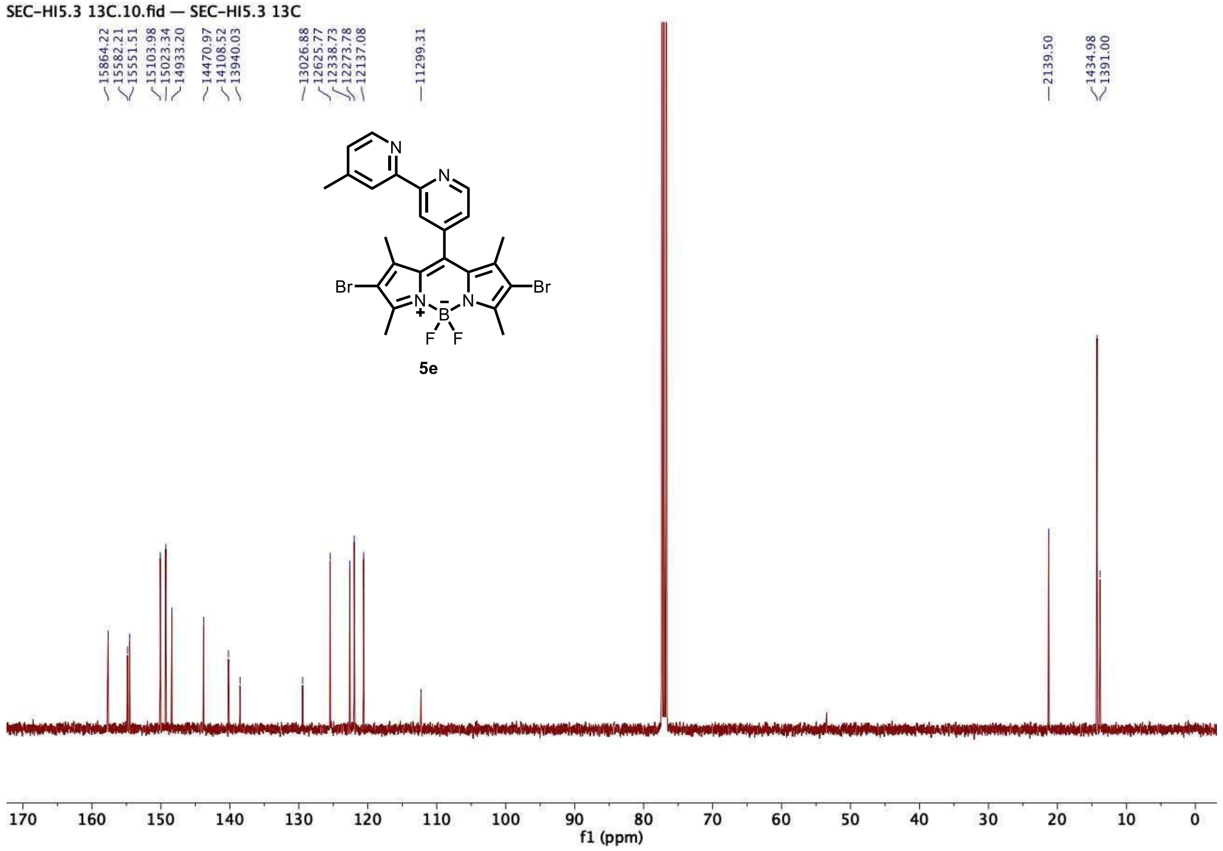

Figure S35. ^{13}C NMR spectra of 5e (100 MHz, $CDCl_3$).

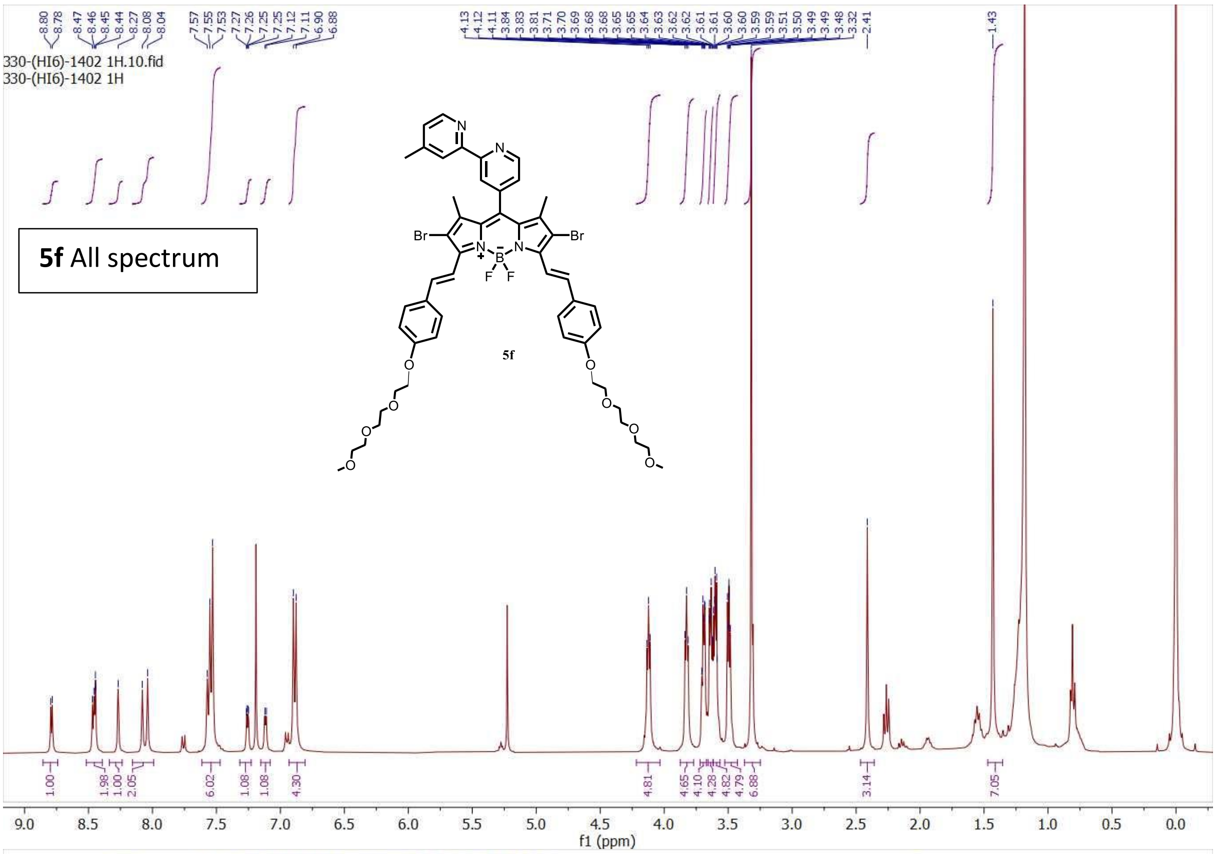
330-(HI6)-1402 1H.10.fid
330-(HI6)-1402 1H
5f All spectrum
5f
f1 (ppm)

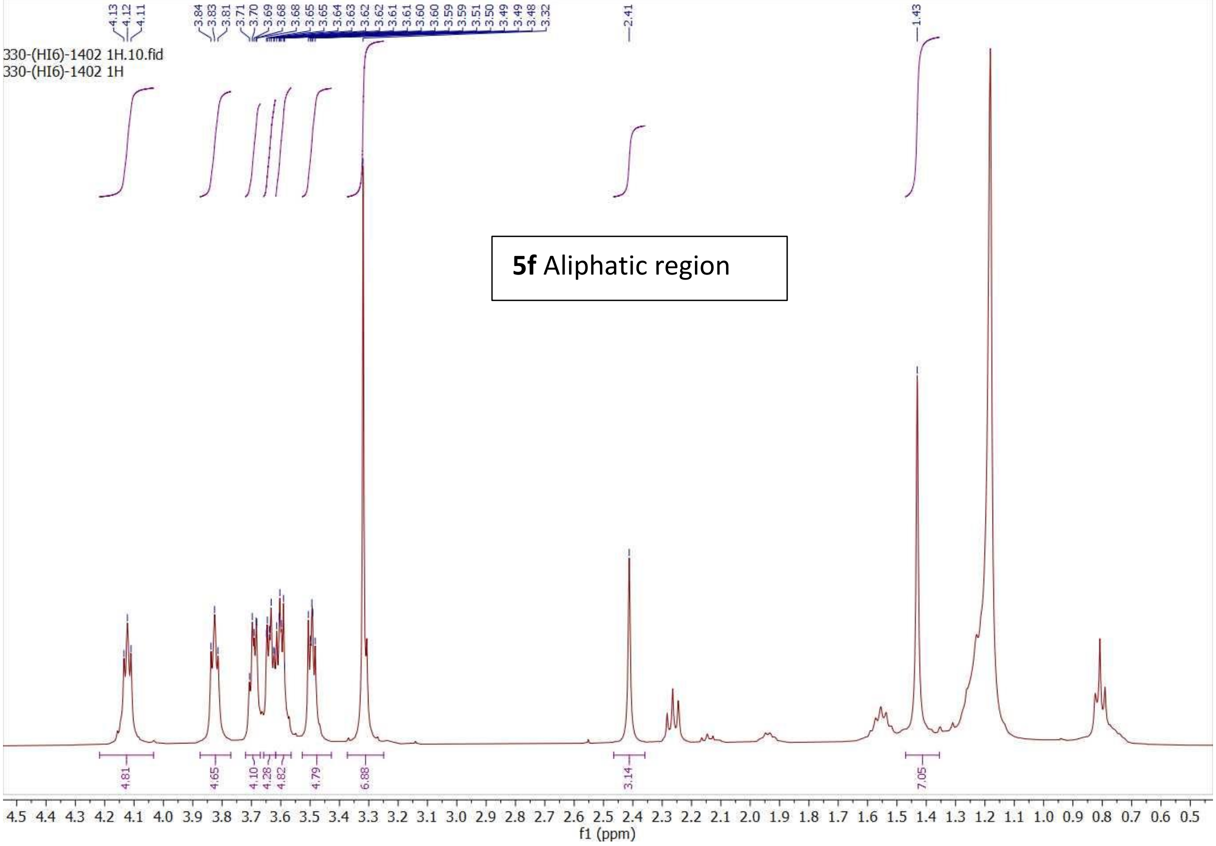
330-(HI6)-1402 1H.10.fid
330-(HI6)-1402 1H
5f Aliphatic region
f1 (ppm)

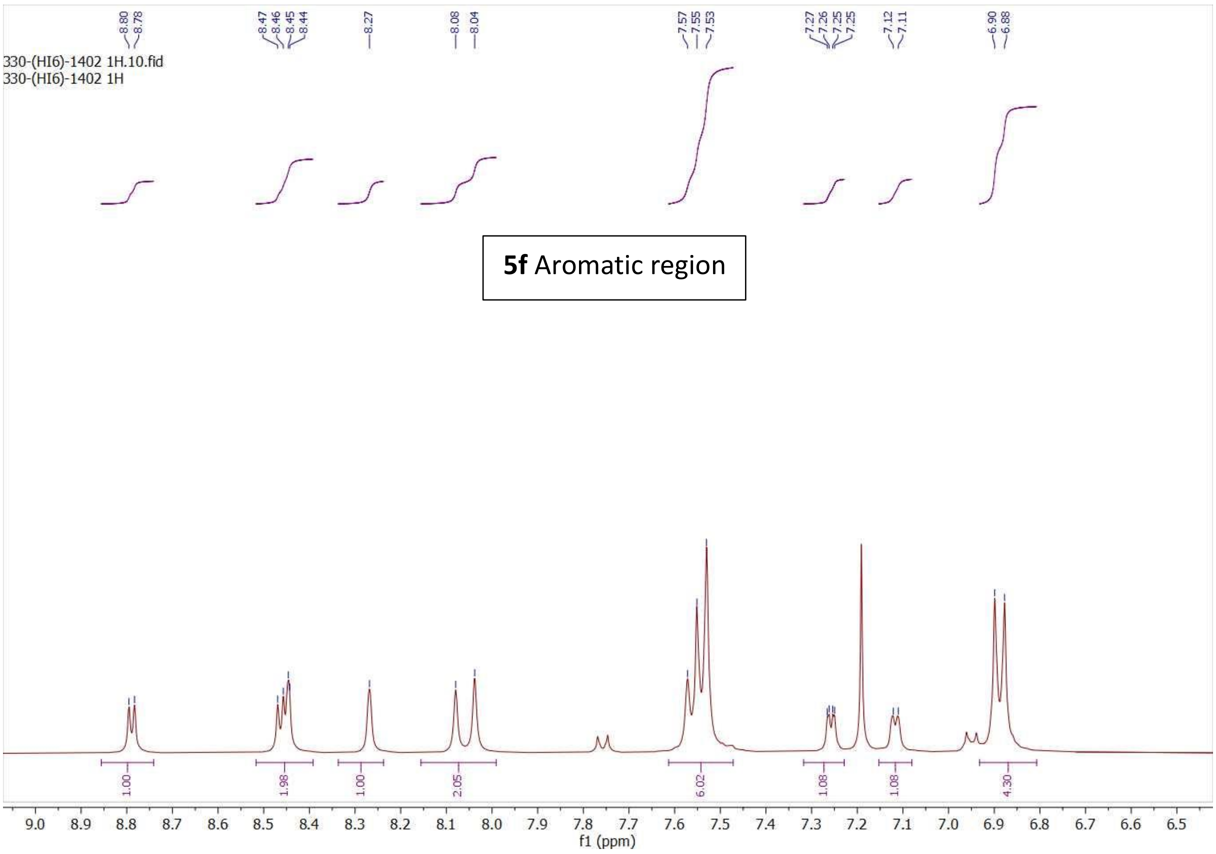

Figure S36. ^{1}H NMR spectra of 5f (400 MHz, $CDCl_3$).

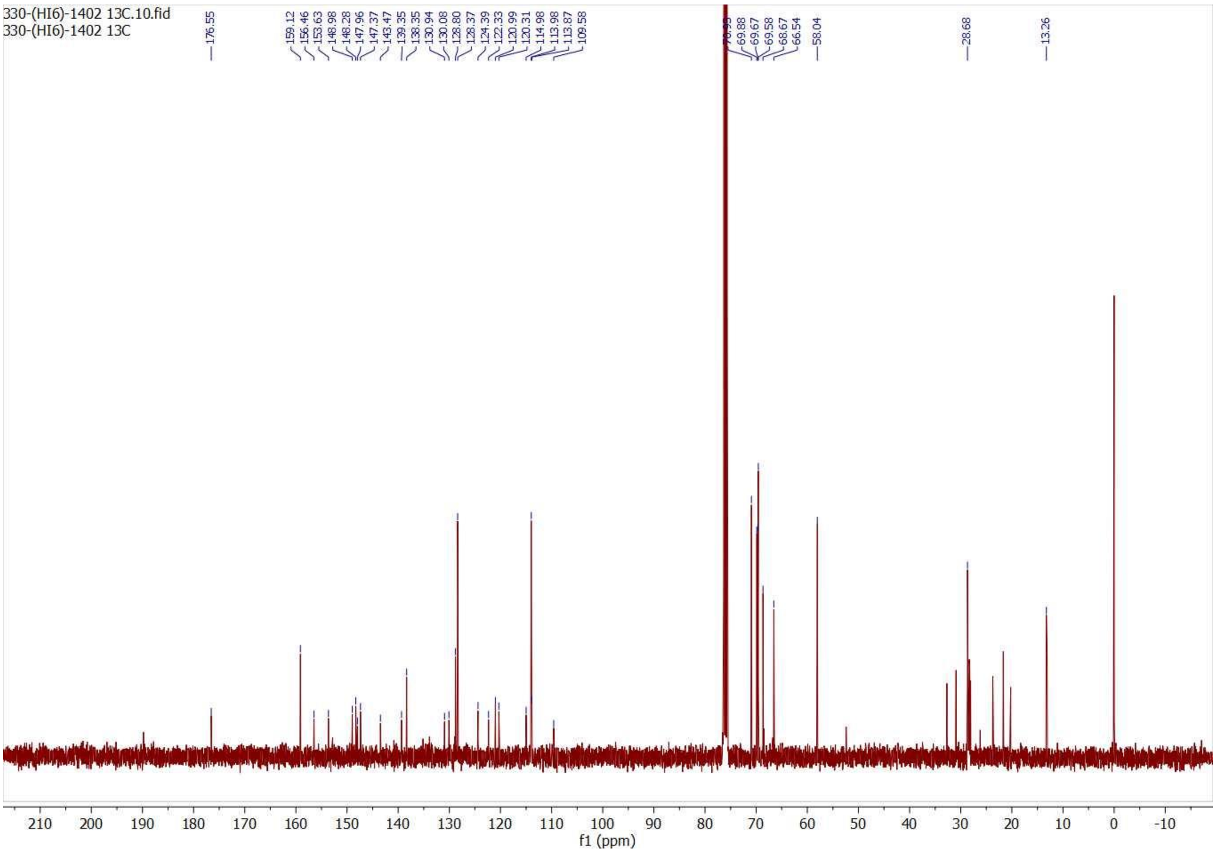

Figure S37. ^{13}C NMR spectra of 5f (100 MHz, $CDCl_3$).

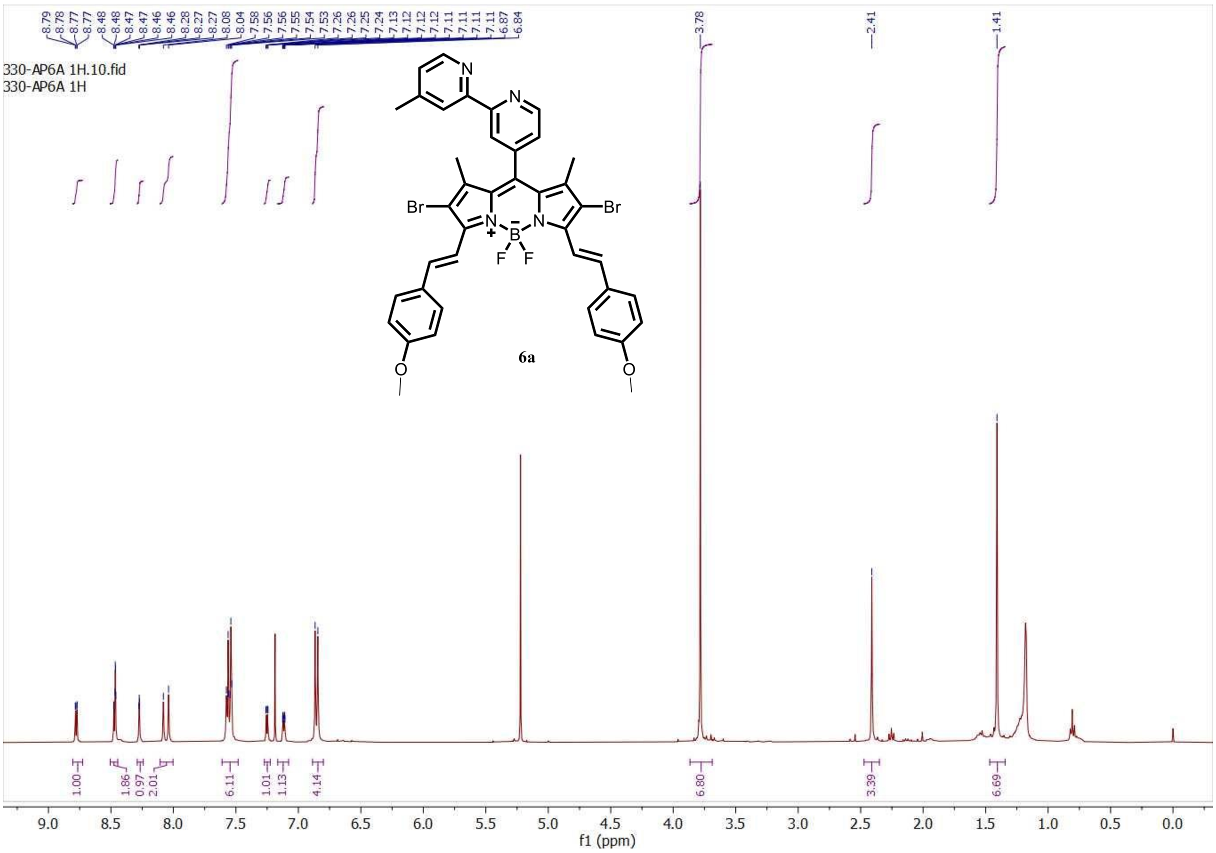

Figure S38. ^{1}H NMR spectra of 6a (400 MHz, $CDCl_3$).

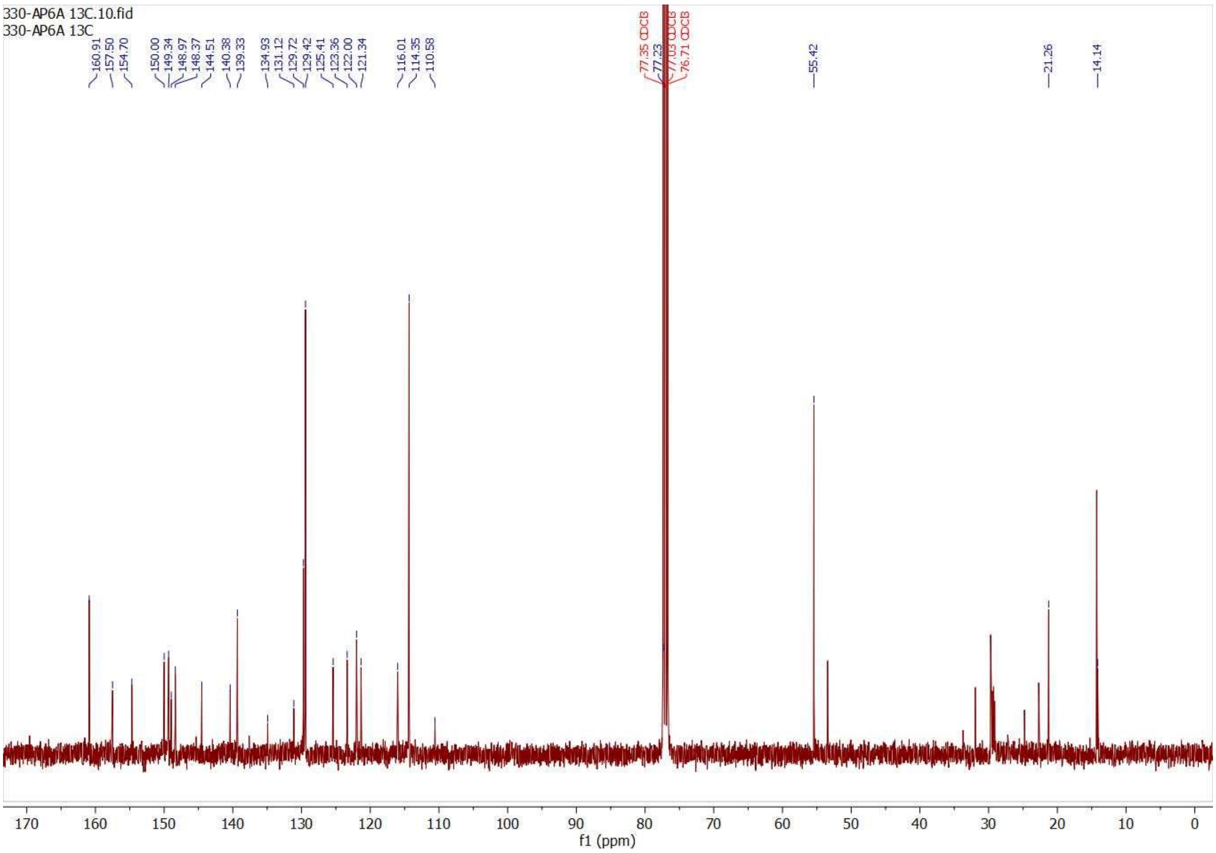

Figure S39. ^{13}C NMR spectra of 6a (100 MHz, $CDCl_3$).

5 High Resolution Mass Spectrometer Data

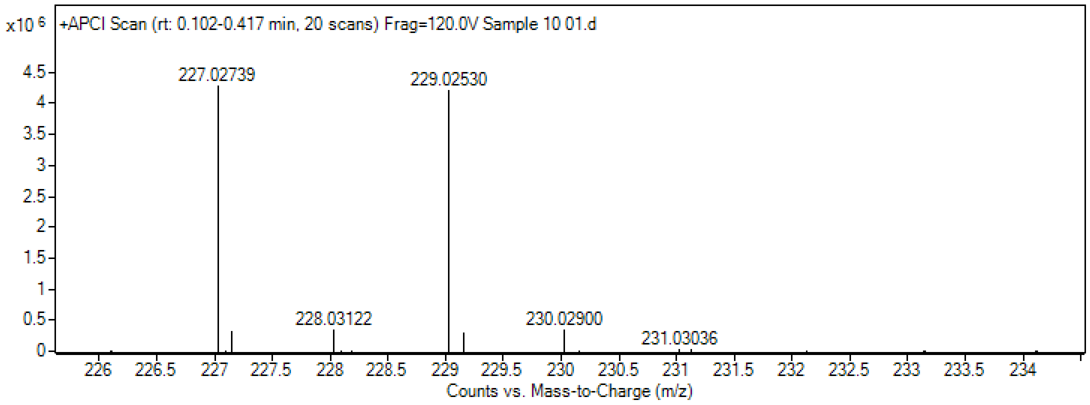

Figure S40. High Resolution ESI-MS spectrum of 5a.

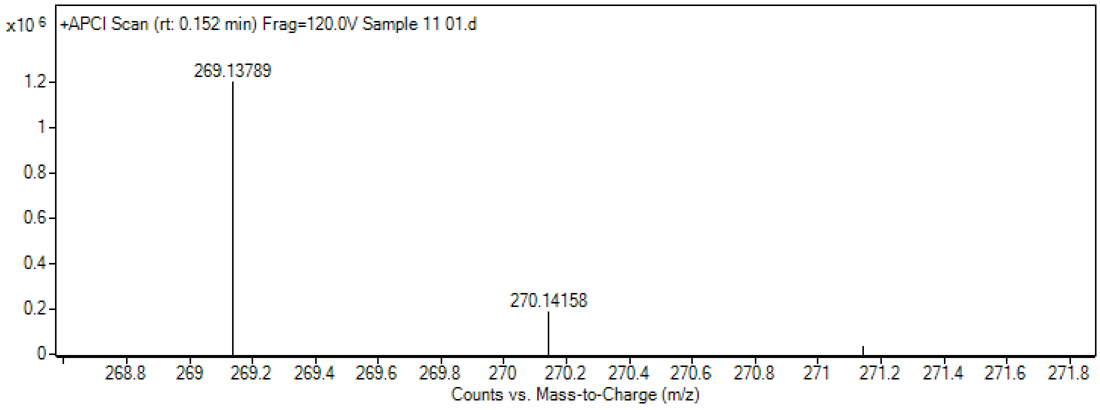

Figure S41. High Resolution ESI-MS spectrum of 5b.

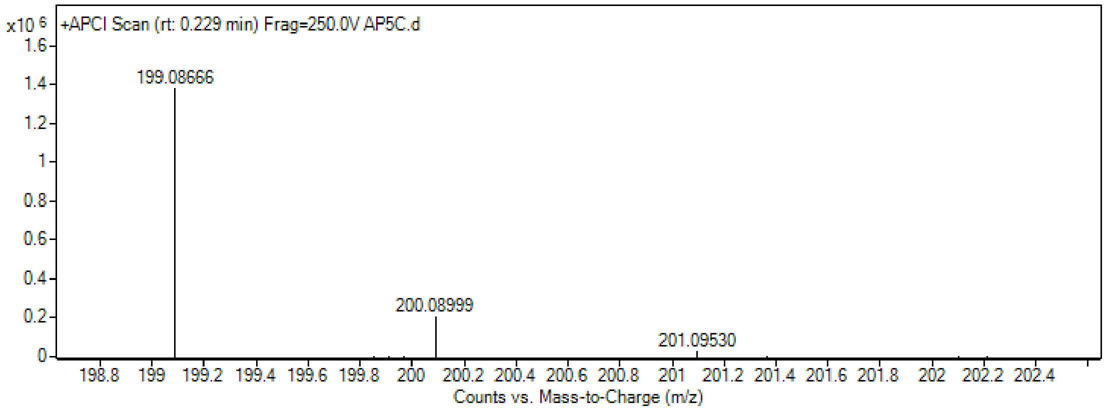

Figure S42. High Resolution ESI-MS spectrum of 5c.

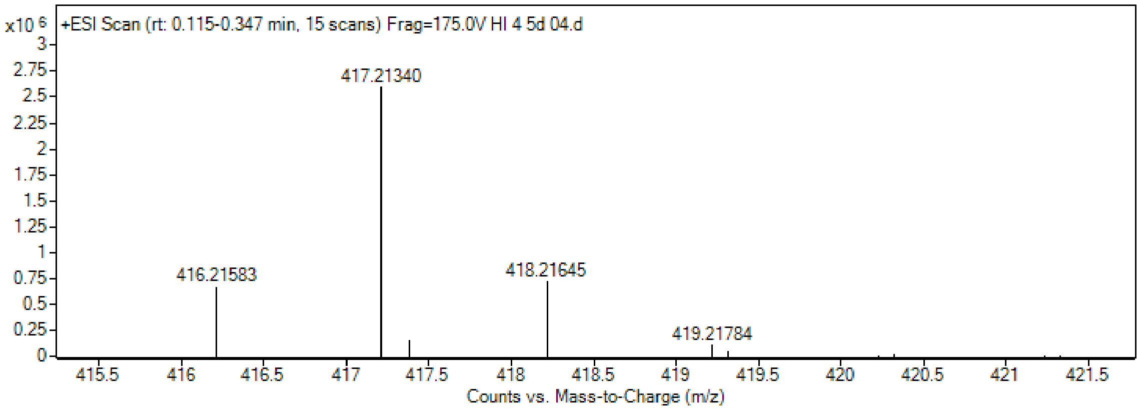

Figure S43. High Resolution ESI-MS spectrum of 5d.

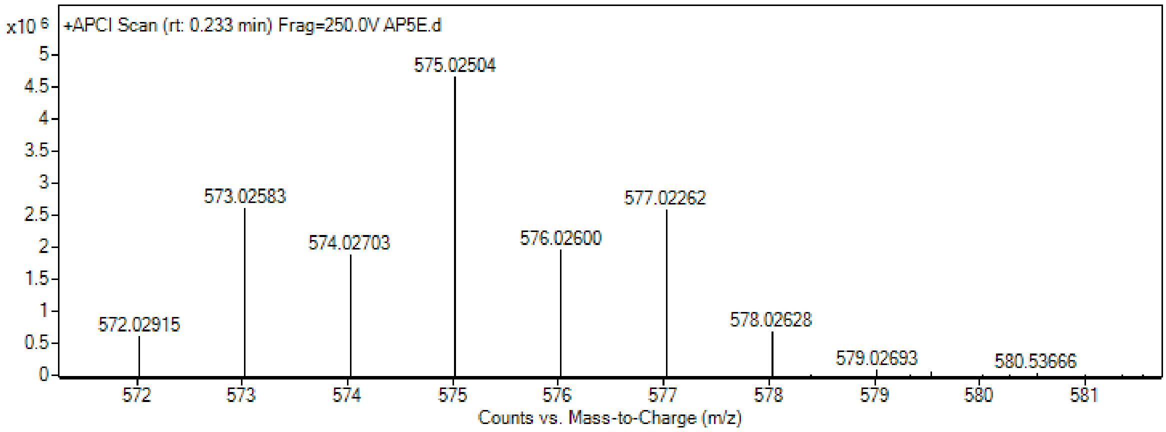

Figure S44. High Resolution ESI-MS spectrum of 5e.

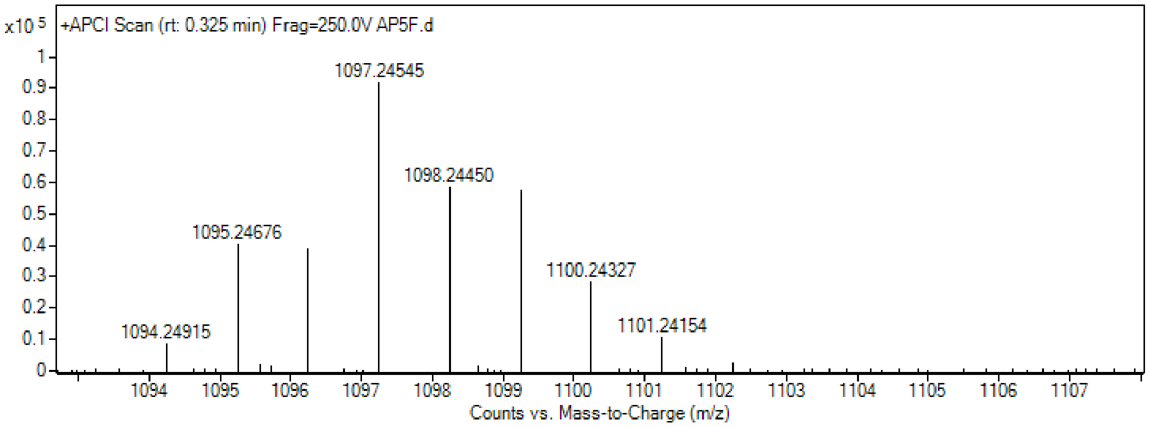

Figure S45. High Resolution ESI-MS spectrum of 5f.

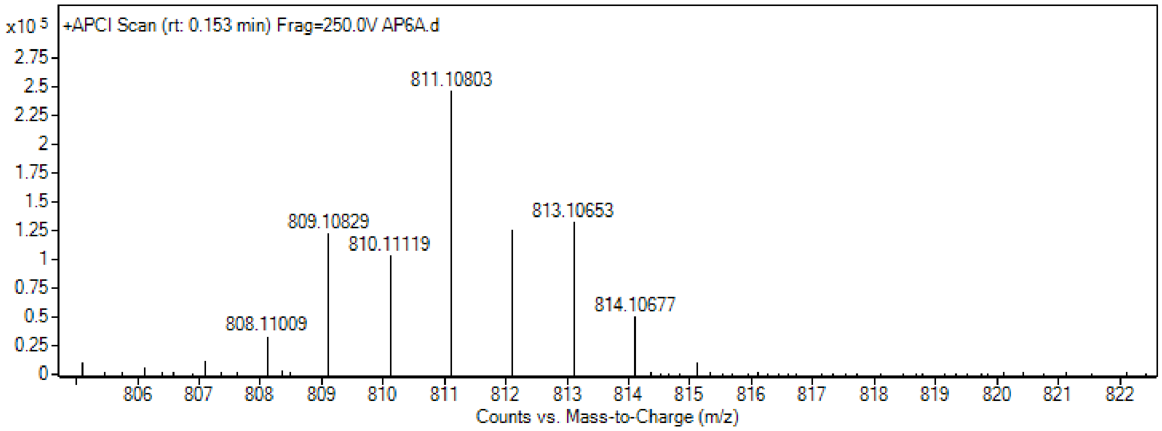

Figure S46. High Resolution ESI-MS spectrum of 6a.

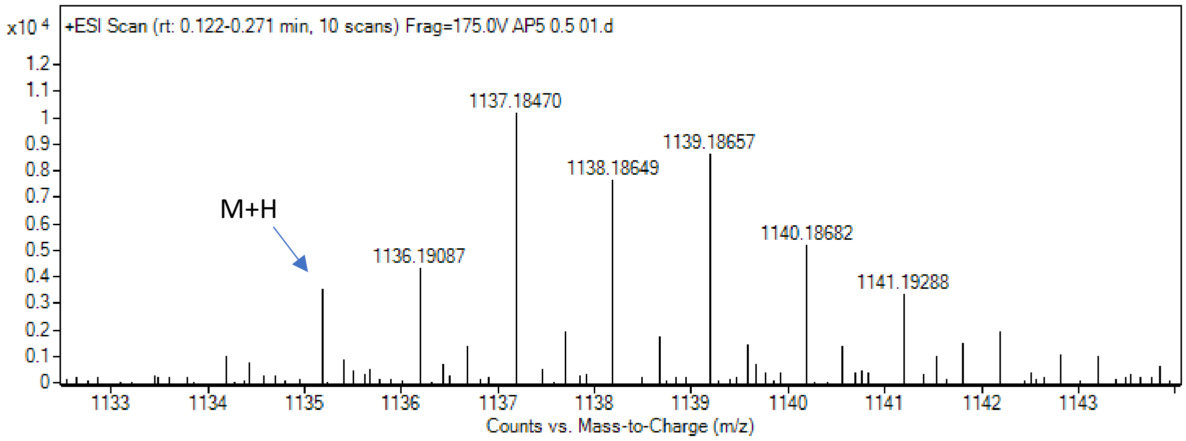

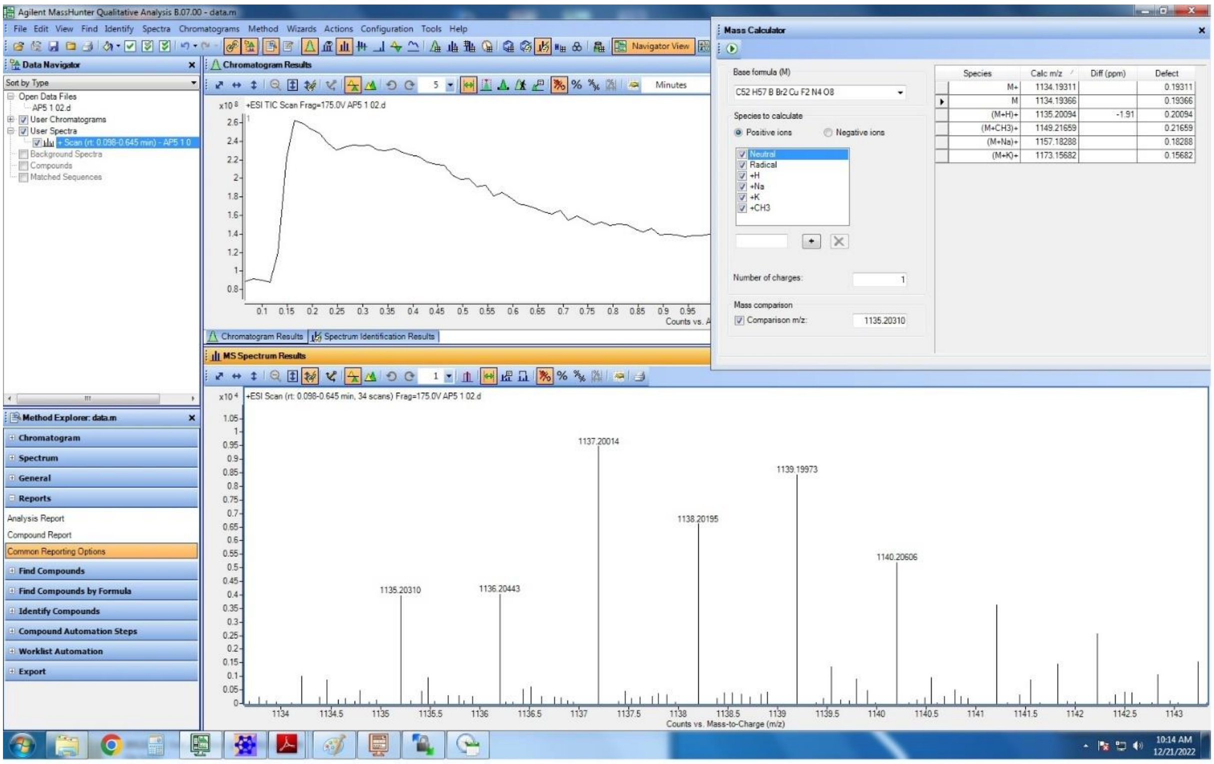

Figure S47. High Resolution ESI-MS spectrum of AP5.

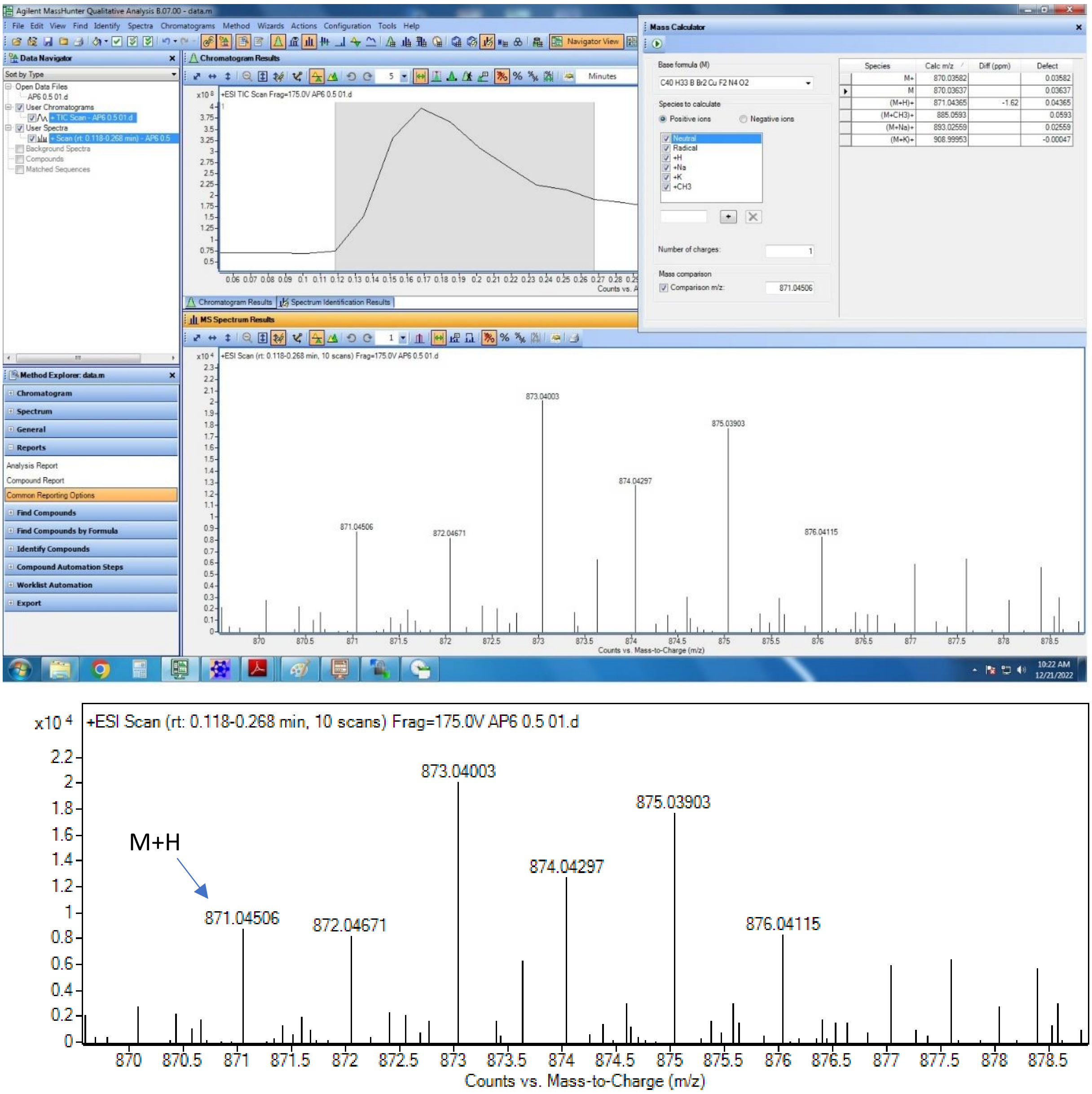

Figure S48. High Resolution ESI-MS spectrum of AP6.

Chromatogram and Results

Injection Details

Injection Name:	**5f**	*Run Time (min):*	**20.00**
Vial Number:	**RA2**	*Injection Volume:*	**2.00**
Injection Type:	**Unknown**	*Channel:*	**UV_VIS_4**
Calibration Level:		*Wavelength:*	**650**
Instrument Method:	**YUSUF HOCA-2**	*Bandwidth:*	**4**
Processing Method:		*Dilution Factor:*	**1.0000**
Injection Date/Time:	**23/Feb/24 14:51**	*Sample Weight:*	**1.0000**

Chromatogram

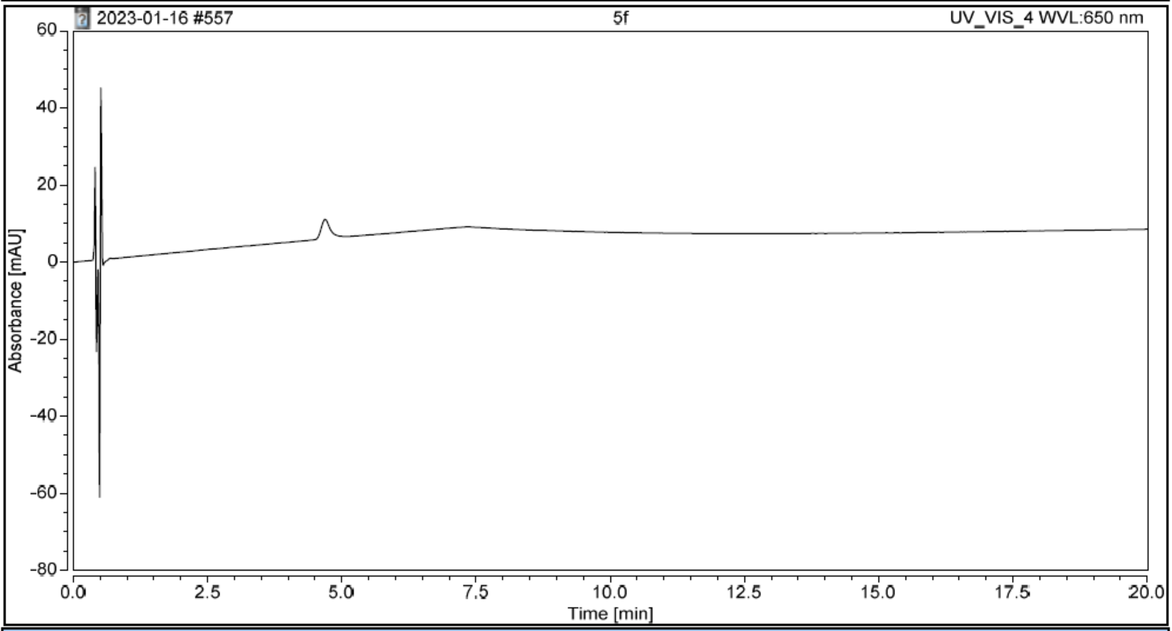

Figure S49. HPLC chromotogram of compound **5f**.

Chromatogram and Results

Injection Details

Injection Name:	6a	*Run Time (min):*	20.00
Vial Number:	RA3	*Injection Volume:*	2.00
Injection Type:	Unknown	*Channel:*	UV_VIS_4
Calibration Level:		*Wavelength:*	650
Instrument Method:	YUSUF HOCA-2	*Bandwidth:*	4
Processing Method:		*Dilution Factor:*	1.0000
Injection Date/Time:	23/Feb/24 15:34	*Sample Weight:*	1.0000

Chromatogram

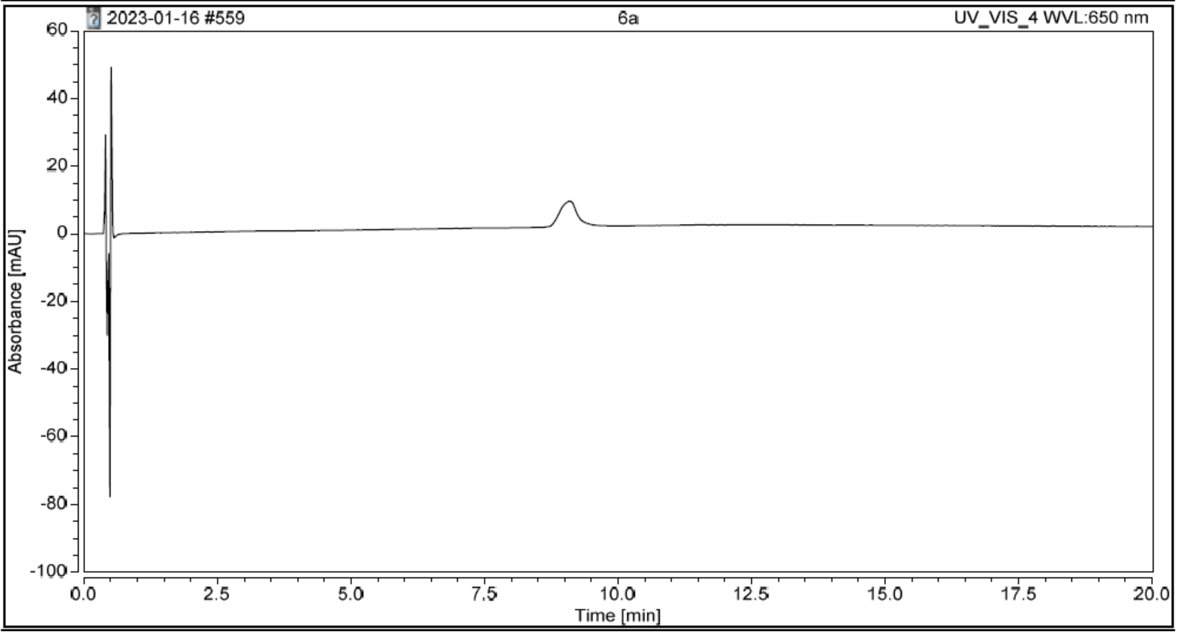

Figure S50. HPLC chromotogram of **6a**.

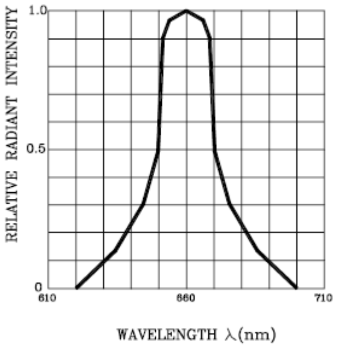

Figure S51. Irradiation wavelength of the LED used in the irradiation experiments (Bright LED, BL-BD03R4V-1)

6 References

(1) Mirloup, A.; Leclerc, N.; Rihn, S.; Bura, T.; Bechara, R.; Hebraud, A.; Leveque, P.; Heiser, T.; Ziessel, R. A deep-purple-grey thiophene-benzothiadiazole-thiophene BODIPY dye for solution-processed solar cells. *New Journal of Chemistry* **2014**, *38* (8), 3644-3653, Article. DOI: 10.1039/c4nj00294f.

(2) Tok, M.; Say, B.; Dölek, G.; Tatar, B.; Özgur, D.; Kurukavak, Ç.; Kus, M.; Dede, Y.; Çakmak, Y. Substitution effects in distyryl BODIPYs for near infrared organic photovoltaics. *Journal of Photochemistry and Photobiology A-Chemistry* **2022**, *429*, 113933. Article. DOI: 10.1016/j.jphotochem.2022.113933.

(3) Can, B.; Çakmak, Y. Exploration of Two Different Strategies in Near IR Absorbing Boron Dipyrromethene Derivatives for Photodynamic and Bioimaging Purposes. *ChemistrySelect* **2021**, *6* (33), 8855-8860, Article. DOI: 10.1002/slct.202102508.

(4) Liu, P.; Li, B.; Zheng, J.; Liang, Q.; Wu, C.; Huang, L.; Zhang, P.; Jia, Y.; Wang, S. A novel N-nitrosation-based ratiometric fluorescent probe for highly selective imaging endogenous nitric oxide in living cells and zebrafish. *Sensors and Actuators B-Chemical* **2021**, *329*, 129147. Article. DOI: 10.1016/j.snb.2020.129147.
